# Genomic decoding of breeding history to guide breeding-by-design in rice

**DOI:** 10.1093/nsr/nwad029

**Published:** 2023-02-09

**Authors:** Zhuo Chen, Qingyun Bu, Guifu Liu, Maoqing Wang, Hongru Wang, Huazhao Liu, Xiufeng Li, Hong Li, Jun Fang, Yan Liang, Zhenfeng Teng, Sai Kang, Hong Yu, Zhukuan Cheng, Yongbiao Xue, Chengzhi Liang, Jiuyou Tang, Jiayang Li, Chengcai Chu

**Affiliations:** State Key Laboratory of Plant Genomics, Institute of Genetics and Developmental Biology, Innovation Academy for Seed Design, Chinese Academy of Sciences, Beijing 100101, China; Key Laboratory of Soybean Molecular Design Breeding, Northeast Institute of Geography and Agroecology, Chinese Academy of Sciences, Harbin 150081, China; State Key Laboratory of Plant Genomics, Institute of Genetics and Developmental Biology, Innovation Academy for Seed Design, Chinese Academy of Sciences, Beijing 100101, China; Key Laboratory of Crop Biotechnology Breeding of the Ministry of Agriculture and Rural Affairs, Beidahuang Kenfeng Seed Co., Ltd, Harbin 150090, China; State Key Laboratory of Plant Genomics, Institute of Genetics and Developmental Biology, Innovation Academy for Seed Design, Chinese Academy of Sciences, Beijing 100101, China; Key Laboratory of Soybean Molecular Design Breeding, Northeast Institute of Geography and Agroecology, Chinese Academy of Sciences, Harbin 150081, China; Institute of Botany, Chinese Academy of Sciences, Beijing 100193, China; Key Laboratory of Soybean Molecular Design Breeding, Northeast Institute of Geography and Agroecology, Chinese Academy of Sciences, Harbin 150081, China; Guangdong Laboratory for Lingnan Modern Agriculture and State Key Laboratory for Conservation and Utilization of Subtropical Agro-Bioresources, College of Agriculture, South China Agricultural University, Guangzhou 510642, China; Key Laboratory of Soybean Molecular Design Breeding, Northeast Institute of Geography and Agroecology, Chinese Academy of Sciences, Harbin 150081, China; State Key Laboratory of Plant Genomics, Institute of Genetics and Developmental Biology, Innovation Academy for Seed Design, Chinese Academy of Sciences, Beijing 100101, China; State Key Laboratory of Plant Genomics, Institute of Genetics and Developmental Biology, Innovation Academy for Seed Design, Chinese Academy of Sciences, Beijing 100101, China; University of Chinese Academy of Sciences, Beijing 100049, China; State Key Laboratory of Plant Genomics, Institute of Genetics and Developmental Biology, Innovation Academy for Seed Design, Chinese Academy of Sciences, Beijing 100101, China; State Key Laboratory of Plant Genomics, Institute of Genetics and Developmental Biology, Innovation Academy for Seed Design, Chinese Academy of Sciences, Beijing 100101, China; State Key Laboratory of Plant Genomics, Institute of Genetics and Developmental Biology, Innovation Academy for Seed Design, Chinese Academy of Sciences, Beijing 100101, China; State Key Laboratory of Plant Cell and Chromosome Engineering, Institute of Genetics and Developmental Biology, Innovation Academy for Seed Design, Chinese Academy of Sciences, Beijing 100101, China; State Key Laboratory of Plant Genomics, Institute of Genetics and Developmental Biology, Innovation Academy for Seed Design, Chinese Academy of Sciences, Beijing 100101, China; State Key Laboratory of Plant Genomics, Institute of Genetics and Developmental Biology, Innovation Academy for Seed Design, Chinese Academy of Sciences, Beijing 100101, China; State Key Laboratory of Plant Genomics, Institute of Genetics and Developmental Biology, Innovation Academy for Seed Design, Chinese Academy of Sciences, Beijing 100101, China; Hainan Yazhou Bay Seed Laboratory, Sanya 572025, China; State Key Laboratory of Plant Genomics, Institute of Genetics and Developmental Biology, Innovation Academy for Seed Design, Chinese Academy of Sciences, Beijing 100101, China; Guangdong Laboratory for Lingnan Modern Agriculture and State Key Laboratory for Conservation and Utilization of Subtropical Agro-Bioresources, College of Agriculture, South China Agricultural University, Guangzhou 510642, China; University of Chinese Academy of Sciences, Beijing 100049, China; Hainan Yazhou Bay Seed Laboratory, Sanya 572025, China

**Keywords:** rice, breeding-by-design, breeding history, genome-wide association study, genomic decoding

## Abstract

Deciphering the intrinsic molecular logic of empirical crop breeding from a genomic perspective is a decisive prerequisite for breeding-by-design (BbD), but remains not well established. Here, we decoded the historical features of past rice breeding by phenotyping and haplotyping 546 accessions covering the majority of cultivars bred in the history of Northeast China (NEC). We revealed that three groups founded the genetic diversities in NEC rice with distinct evolution patterns and traced and verified the breeding footprints to known or genome-wide association study (GWAS)-detected quantitative trait loci (QTLs), or introgressions from *indica* sub-species with chronological changes in allele frequencies. Then we summarized a rice breeding trend/principle in NEC, and combined with the successful example in breeding and application of Zhongkefa5 to demonstrate the guiding value of our conclusion for BbD in practice. Our study provides a paradigm for decoding the breeding history of a specific crop to guide BbD, which may have implications in different crop breeding.

## Teaser

The genomic decoding of rice breeding history in Northeast China, along with the successful example in breeding of Zhongkefa5, shows guiding value for rice breeding-by-design (BbD) and may have implications in different crop breeding.

## INTRODUCTION

With the continuous progress of functional genomic research, the concept of breeding-by-design (BbD) was proposed and put into practice to breed cultivars in a rapid, precise and systematic manner [1–4]. Generally, the design scheme will be established based on the current knowledge of the functional dissection of agronomic traits [5], which is essentially a process for purposefully combining a series of favorable allelic variations that are usually dispersed in different genetic resources, or designed allelic variations that not present in natural populations, for genes of agronomic importance. However, breeding based solely on the functional information of multiple genes themselves is impractical. There are at least two implicit prerequisites for the successful implementation of this process, namely the selection of backbone parents and the matching of characteristics of target cultivars with the environment and planting conditions in the planting region and the preferences of consumers. The former is the starting platform of design breeding and in most cases includes major products of past breeding, and the latter determines the promotion and application of the final bred cultivar. The setting of both is actually inseparable from a comprehensive understanding of the intrinsic logic of past breeding practice. How to interpret the intrinsic rules from the functional genomics perspective and integrate them into BbD practice is one of the key but not well-elucidated fields.

Breeding objectives for crops always have historical and regional features that largely depend on new cultivation techniques, market demand and commercial value for the end products of crops, and the fluctuating environments including climatic changes and pathogen/insect evolvement [6]. For example, the popularization of cultivation techniques has led to the selection of varieties adapted to direct seeding [7] and the improvement in the timeliness of harvesting and storage has spawned new requirements for low grain moisture content at harvest [8]. In addition, with the improvement in people's quality of life, food smell and taste are also changing and required [4,9]. All these have imperceptibly led breeders to flexibly tune their breeding directions. Therefore, the analyses from the perspective of changes in the times will be helpful to better illustrate the past breeding logic for a specific region.

Rice is grown in almost every province of China. Among them, rice breeding in Northeast China (NEC) covered by Heilongjiang (HLJ), Jilin (JL) and Liaoning (LN) provinces has attracted more attention due to the following facts. NEC is the northern limits (38.7–53.5°N, latitude) of rice cultivation in China and one of the northern limits of rice cultivation around the world. Owing to its unique natural and geographical conditions, NEC is an important region producing *japonica* rice with high grain quality in China. As the excellent eating quality of rice caters to the growing demands of consumers, the rice planting area and grain production in NEC have also increased substantially over the past few decades [10]. Notably, HLJ has been in a stable position as the largest rice production province in China (National Bureau of Statistics of China, NBSC, http://www.stats.gov.cn/enGliSH/). Since the planting area has reached the upper limit, strengthening and accelerating the breeding of new elite varieties is the best way to maintain the dominant position of the region in the supply of high-quality rice. Moreover, while rice cultivation in South and Southeast China can be traced to thousands of years ago, large-scale rice cultivation was introduced to NEC by Korean migrants only in the nineteenth century [11,12]. The short breeding history makes it possible to establish a basic decoding model of breeding logic that considers the chronological characteristics of breeding.

Based on these, we collected and resequenced a panel of 546 rice cultivars bred in NEC or imported from Korea and Japan (Supplementary Table S1). By integrating phenotypic, genomic and historical clues, we disclosed the landscape of genetic variations in NEC rice collection, providing informative insights regarding agronomical traits, pedigrees, introgressions and quantitative trait loci (QTLs). Finally, we summarized a trend/principle of rice breeding in NEC and demonstrated its potential guiding value in rice BbD in combining with the successful breeding of Zhongkefa5 (ZKF5).

## RESULTS

### Chronological features of rice cultivars in NEC

**Figure 1.**
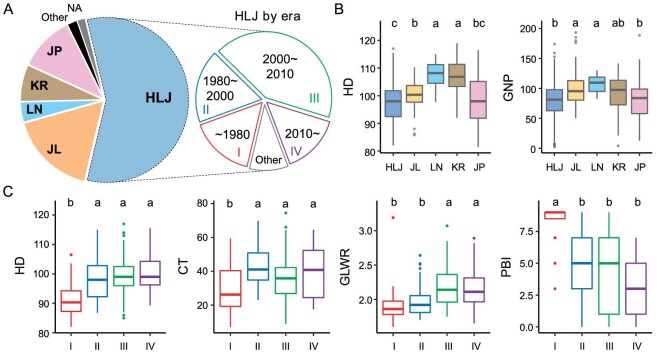
Geographical distribution and phenotypic divergence of rice in NEC. (A) Geographic and chronological distribution of cultivars in this study. HLJ, Heilongjiang province; JL, Jilin province; LN, Liaoning province; KR, Korea; JP, Japan. (B) Phenotypic variation of rice heading date (HD) and grain number per panicle (GNP) among different regions. (C) Phenotypic variation of rice in HLJ bred in different eras. CT, seed-setting rate after cold treatment; GLWR, grain length/width ratio; PBI, panicle blast index. Multiple comparison was performed by using Tukey's honestly significant difference (HSD) test.

Modern rice breeding in NEC began with the introduction of few rice varieties from Japan and Korea in the 1940s, which subsequently became the backbone parents for local pedigree breeding and hybrid breeding [13,14]. To explore the patterns of genetic diversity within the rice cultivars in NEC, we collected and resequenced 546 cultivars (Supplementary Table S1) from the three provinces in NEC as well as cultivars from Korea and Japan (Fig. 1A), which represent the vast majority of introduced or bred cultivars in NEC from the 1940s to the 2010s. HLJ is the largest rice production province in China, accounting for ∼72% of the total rice production in NEC (NBSC, http://www.stats.gov.cn/enGliSH/). Accordingly, over half (309/546) of the cultivars in this collection were from HLJ. To more intuitively show the chronological features of the phenotypic and genomic changes of rice cultivars in HLJ, we further divided the HLJ cultivars into four historical groups (Fig. 1A), namely HLJ-I (∼1980), HLJ-II (1980–2000), HLJ-III (2000–10) and HLJ-IV (2010∼), by considering the representativeness determined by population size and the fact that the number of newly bred rice varieties exploded after 2000.

Phenotype data for 22 agronomic traits were measured in Experimental Station of Chinese Academy of Sciences (45.7°N, 126.6°E) in Harbin city of HLJ, during the natural growing season (Supplementary Table S2). Cultivars from different regions showed divergent phenotype, in correspondence with the growing conditions and preferences of the breeders. Cultivars from JL (40.9–46.3°N, latitude) and LN (38.7–43.4°N, latitude) had a later heading date and a higher grain number per panicle, which enable them to obtain higher yields in lower latitude regions with relatively abundant light and accumulated temperature compared with that in HLJ (43.4–53.5°N, latitude) (Fig. 1B). Indeed, cultivars from HLJ show generally earlier heading to harvest a reasonable yield before cold winter in higher latitudes (Fig. 1B). However, it is intriguing to observe a trend of delayed heading date in HLJ cultivars after 1980, which may benefit from warming climate [15] and the promotion of greenhouse seedling technology [16], making it possible to obtain higher yield by prolonging the growth period (Fig. 1C). Delayed flowering increased the risk of chilling damage during reproductive growth, also driving the improved chilling tolerance of cultivars (Fig. 1C). We also observed a trend for increasing grain length/width ratio (GLWR) and panicle blast resistance over time in cultivars from HLJ, which may have resulted from the growing preference of consumers for slender grain rice and increasing threat of rice blast disease (Fig. 1C).

### NEC rice cultivars can be traced to three subgroups

**Figure 2.**
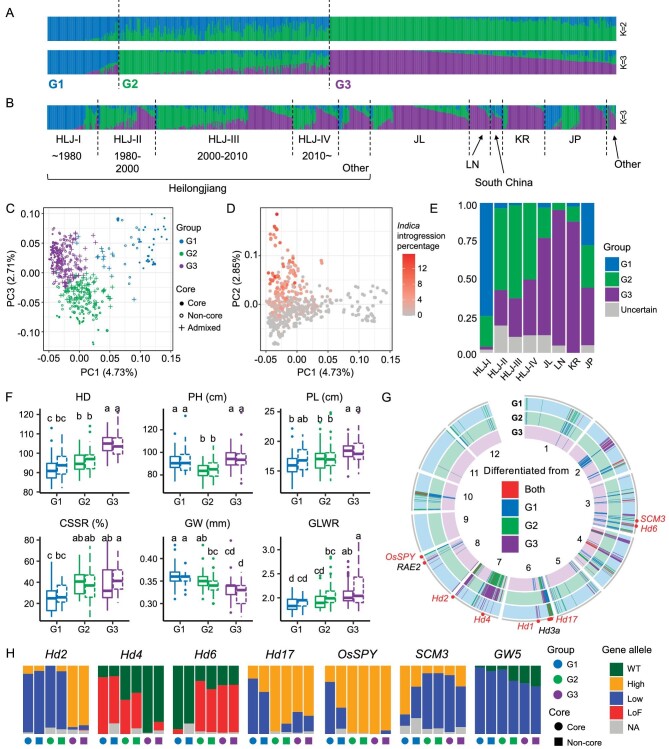
Three rice subgroups founded the genetic diversities in NEC rice with distinct evolution patterns. (A) and (B) Subgroups inferred by using Admixture software assuming two (K = 2) and three ancestries (K = 3) of 546 cultivars using genome-wide SNP markers. Samples are sorted by ancestry scores (A) and by geographical origin when K = 3 (B). HLJ, Heilongjiang province; JL, Jilin province; LN, Liaoning province; KR, Korea; JP, Japan. (C) and (D) Principal component analysis (PCA) of 546 cultivars using SNP markers; samples are colored by the subgroups inferred by using Admixture assuming three ancestries (G1, G2 and G3) (C) and by the percentage of introgressions from *indica* sub-species (D). (E) Proportion of cultivars from the three subgroups (G1, G2 and G3) in different regions and eras. (F) Phenotypic variation of four traits among the three subgroups (G1, G2 and G3) inferred by using Admixture. Solid line for core cultivars and dashed line for non-core cultivars. HD, heading date; PH, plant height; PL, panicle length; GW, grain width; GLWR, grain length–width ratio; CSSR, seed-setting rate after cold treatment. Multiple comparison performed by using Tukey's HSD test. (G) Genome distribution of highly differentiated (*F*_ST_ top 5%) regions between each of the two subgroups. (H) Allele frequency of important differentiated QTLs among subgroups. In each group, core cultivars (ancestry score > 0.99) and non-core cultivars (ancestry score ≤ 0.99) are tabulated separately. Gene alleles are defined as follows: WT, functional wild-type; High, higher protein function or expression level; Low, lower protein function or expression level; LoF, loss-of-protein function.

To dissect genomic changes of rice cultivars in NEC, all cultivars collected were sequenced at least five times (Supplementary Table S1). While it has been clear that most rice cultivars in NEC belong to the temperate-*japonica* subpopulation [17], we sought to gain a more comprehensive knowledge of the population structure and genetic make-up of this rice collection with genome-wide markers. First, we performed genome-wide subpopulation ancestry inference using single nucleotide polymorphism (SNP) markers [17] derived from the 3K-RG project [18]. As inferred by 3K-RG markers, all NEC rice cultivars were inferred as from temperate-*japonica* (Supplementary Table S3). Population structure inference was performed by using Admixture [19] software, identical-by-state (IBS) analysis and principal component analysis (PCA) (Supplementary Table S3). According to the inference of Admixture, the cross-validation error continued to decrease as the number of assigned ancestries (K) increased (Supplementary Fig. S1A); we therefore took the combination of Admixture and IBS analysis to determine a proper grouping. When assigning two ancestries (K = 2), the population was separated into two subgroups: K2G1 (Group 1 when assigning two ancestries) and K2G2 (Fig. 2A). When K = 3, the former K2G2 group was further divided into two groups: K3G2 and K3G3. When K = 4, a new group K4G4 that consists of cultivars from both K3G2 and K3G3 emerged (Supplementary Fig. S1B), but the mean dissimilarity among cultivars within K4G4 exceeded that between K4G4 and K4G3, indicating an unproper grouping (Supplementary Fig. S1C). Therefore, we found that it is reasonable to separate the population into three subgroups (Fig. 2B and Supplementary Table S3). We reperformed the Admixture analysis 100 times with subsets of SNP markers and found that all the replicates supported the original grouping with the total data set when K = 3. The grouping became highly unstable with K > 3 (Supplementary Fig. S1D). When K = 3, 56 samples were assigned to different subgroups in >25 replicates and were considered to be unreliably grouped. These 56 samples were not included in analysis based on the three subgroups. A neighbor-joining tree constructed using the same SNP data set also supported the divergence of the inferred subgroups (Supplementary Fig. S1E). PCA showed that the population structure was weak, with the top 10 principal components (PCs) explaining only 23.49% of the total variation (Supplementary Table S3). Among the top three PCs, PC1 and PC3 supported the clustering of the three subgroups inferred by using Admixture (Fig. 2C), while PC2 was highly correlated with the proportion of *indica* components in the cultivars (*r*^2^ = 0.545, *P*-value = 8.57 × 10^−95^), indicating a substantial impact of *indica* introgression on the genetic structure ofNEC *japonica* cultivars (Fig. 2D).

Known backbone cultivars were found within the core cultivars (ancestry score > 0.99) of the three subgroups (Supplementary Table S3). For example, the core cultivars of K3G1 included a well-known Japanese cultivar, Ishikari-Shiroge, which was developed in the high-latitude region Hokkaido. Over 70% of the cultivars from HLJ before the 1980s were clustered within K3G1 (Fig. 2E), consistently with the record that Ishikari-Shiroge was introduced to HLJ in the 1940s and since then has served as the backbone breeding material for decades [13]. Core cultivars of K3G2 included backbone cultivars from Japan, such as Yukara and KuiKu131 (Kongyu131 in China, KY131). K3G3 included backbone cultivars such as Fujihikari, Akihikari and Fukei138. These results collectively indicate the fundamental contribution of *japonica* backbone pedigrees to the genetic structure of NEC rice.

The distribution of the three subgroups was uneven in different regions and eras (Fig. 2E). In HLJ, K3G1 was dominant in cultivars before the 1980s, but was replaced by K3G2 thereafter. K3G3 was dominant in JL and LN, and with increasing proportions in HLJ over time. The phenotypic differentiation of the three major subgroups corresponded to their geographical and chronological distribution (Fig. 2F). Group K3G1 was characterized by extremely early flowering that may be the key to their successful adaptation to high-latitude HLJ in the early years. In contrast, with the improvement in the cultivation experience and farming techniques, cultivars in K3G2 showed relatively later flowering than K3G1, but lower plant height and stronger cold tolerance (Fig. 2F). K3G3 showed even later flowering than K3G2 and with higher plant and longer panicle (Fig. 2F). Also, the grain width decreased from K3G1 to K3G3 and the GLWR increased accordingly (Fig. 2F).

### Breeding traces of NEC rice cultivars are linked to major QTLs

The phenotypic differentiation between the subgroups should be caused by genetic variation. We performed genome-wide scan for genetic differentiation (*F*_ST_) in a pairwise manner among the three subgroups (Fig. 2G and Supplementary Fig. S2). The top differentiated regions (top 5% in each comparison) harbored several known QTLs for heading, awn and plant architecture (Fig. 2G and Supplementary Table S4). Among them, *Hd2* and *Hd4* were reported to be the major determinants of the heading date variation in NEC [20]. The weak allele (allele with low effect) of *Hd2* confers earlier flowering [21,22] and was nearly fixed in K3G1 and K3G2, while the strong allele was dominant in K3G3 (Fig. 2H). A similar pattern was also found on *Hd4* as its loss-of-function (LoF) allele [23] presents mainly in K3G1 (Fig. 2H). The weak allele of *OsSPY* confers higher plant height [24] and was present mainly in K3G1 but was absent in K3G2, which may explain the difference in plant height between these two populations (Fig. 2H). The strong allele of *SCM3*/*OsTB1* on chromosome 3 was reported to confer strong culm and bigger panicle but lower tiller number [25], and was present mainly in K3G1 (Fig. 2H). Interestingly, while they are located on different chromosomes, the weak culm and tiller promoting allele of *SCM3* was linked to the short culm allele of *OsSPY* in K3G1 (Chi-square test Phi = 0.56; *P*-value = 9.44 × 10^−5^). This linkage indicates a potential co-selection for lodging resistance and tillering in *japonica* rice, just like the relationship between *SD1* and *HTD1* [26] in *indica* rice for the Green Revolution. Some other QTLs that did not fall within the top differentiated regions also showed diverged allele distribution among the subgroups. The weak allele of *GW5* [27] in *japonica* rice cultivars contributed to wider grain and the higher ratio of wild-type allele in G2 and G3 (Fig. 2H) may account for their slender grain compared with G1 (Fig. 2F).

**Figure 3.**
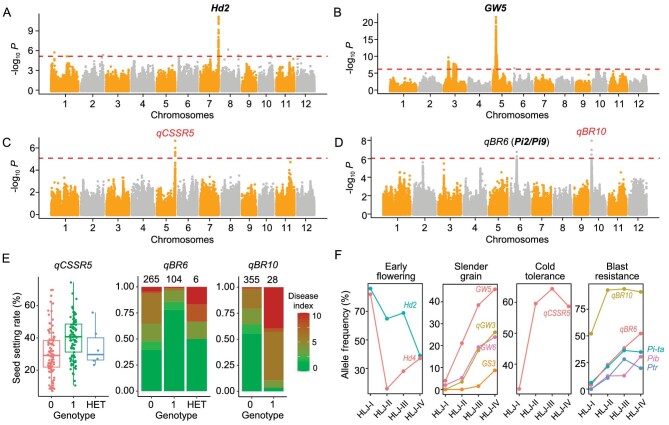
Known and novel QTLs detected by using genome-wide association studies with chronological changes in allele frequencies. (A)–(D) Manhattan plots of genome-wide association studies on heading date (A), grain width/length ratio (B), seed-setting rate after cold treatment (C) and leaf blast index in 2010 (D). Known QTLs are marked in bold and novel QTLs except *qBR6*, which may be allelic to *Pi2/Pi9*, are marked in red. (E) Phenotype distribution of different alleles on novel QTLs for cold tolerance and blast resistance. On each QTL, genotype 0 for reference genome allele (Nipponbare) and genotype 1 for alternative allele. HET, heterozygous genotypes. (F) Allele frequency of known and novel QTLs that showed chronological trends of improvement in Heilongjiang province.

The effect of a QTL may rely on a certain genetic background and/or experimental conditions; we thus tested the phenotypic effects of these QTLs separately in each of the three subgroups. We genotyped 111 QTLs with functionally verified natural variants or linkage markers (Supplementary Table S5). This analysis yielded a list of QTLs with significant effect on their related traits in NEC (Supplementary Table S6). For example, *Hd2* and *Hd4*, the major contributors of the heading date variation, also showed impacts on the panicle traits. Grain shape was controlled by *GW5* [27], *GS3* [28] and *GW6* [29]. *Pi9* [30] and *Pib* [31] were effective to blast resistance. Multiple QTLs including *SCM2* [32], *SCM3* [25], *DEP1* [33], *LAX1* [34] and *OsSPY* [24] collectively influenced panicle architecture. Some known QTLs were further validated and novel QTLs were detected by using genome-wide association study (GWAS) (Supplementary Fig. S3 and Supplementary Table S7). The known QTLs validated by using GWAS include *Hd2* for heading, *GW5* for grain shape and the Reimei-type semi-dwarf allele of *SD1* [35] (Fig. 3A and B, and Supplementary Fig. S4). A novel QTL for cold tolerance (seed-setting rate after cold treatment at booting stage) was found on chromosome 5, named as *qCSSR5* (Fig. 3C and E). The cold-tolerant allele of this QTL was dominant (>90%) in K3G2 and was also present with a relatively high ratio in the other two subgroups. Two QTLs for rice blast resistance were detected including a novel QTL *qBR10* on chromosome 10 and *qBR6* on chromosome 6, which is allelic to the well-known *Pi2/Pi9/Piz-t/Pigm* [30,36,37] locus (Fig. 3D and E). The susceptible allele of *qBR10* was present mainly in early cultivars and is nearly eliminated in modern cultivars.

By combining the known QTLs with significant phenotypic effect or geographical differentiation signatures, as well as those novel QTLs detected by using GWAS, we assembled a list of applicable QTLs for breeding in NEC (Fig. 5B). The weak allele of *Hd2* leads to earlier heading and presents with high frequency in HLJ but with low frequencies in lower latitudes such as JL, LN and KR. The dense and erect allele of *DEP1* presents with high frequency in LN, which reflects local breeding preference. The Reimei semi-dwarf allele of *SD1* is dominant in Korean cultivars (Supplementary Table S5D). Corresponding to the chronological evolution of agronomic traits in HLJ (Fig. 1C), we detected changes in allele frequencies of QTLs associated with them (Fig. 3F), including a remarkable reduction in the early heading allele of *Hd2*, increasing frequencies of multiple alleles (*GW5, GS3, GW6, qGW3*) conferring slender grain, cold tolerance (*qCSSR5*) and blast disease resistance (*qBR6* and *qBR10*). These QTLs partially explained the genetic basis for increased yield, improved quality and strengthened adaptation to biotic and abiotic stresses in HLJ rice breeding over decades. Such findings will be useful for the choice of basic breeding materials and also marker-assisted selection during BbD in the future.

### Introgressions from *indica* sub-species mainly contribute to yield and resistance traits

**Figure 4.**
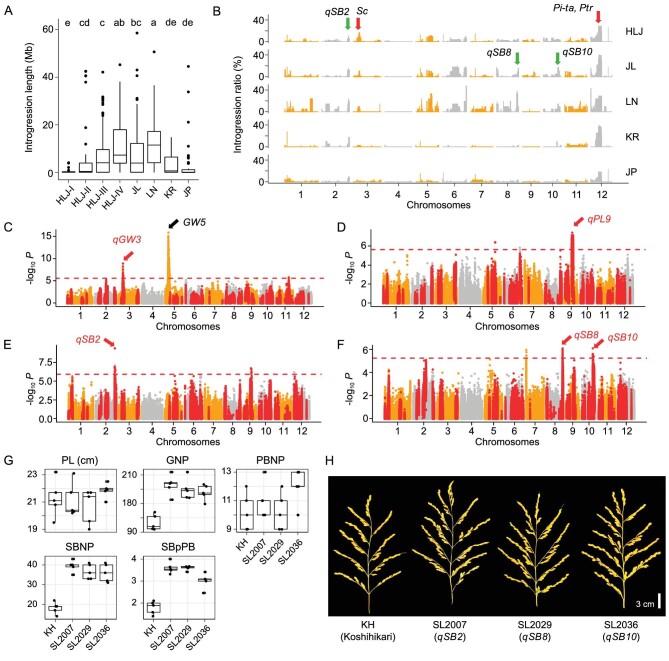
Distribution of *indica*-derived introgressions and their impact on agronomical traits. (A) Percentage of introgressions from *indica* sub-species in cultivars from different regions and eras. Multiple comparison performed by using Tukey's HSD test. (B) Frequency and positional distribution of *indica* introgressions in cultivars from different regions. (C)–(F) Manhattan plots of genome-wide association studies on grain width (C), panicle length (D), secondary branching number per panicle (E) and secondary branching number per primary branching (F). SNPs correlated with *indica* introgressions are highlighted in red. (G) and (H) Panicle phenotypes of *japonica* cultivar Koshihikari (KH) and introgression lines carrying QTLs from *indica* cultivar IR64. PL, panicle length; GNP, grain number per panicle; PBNP, primary branching number per panicle; SBNP, secondary branching number per panicle; SBpPB, secondary branching number per primary branching.

In NEC, almost all rice cultivars belong to temperate-*japonica* with limited genetic background. To further improve the yield and resistance traits, rice breeders broadened the genetic background by introducing *indica* genetic components [38]. These efforts changed the genetic components of NEC rice as clearly reflected in the population structure analysis (Fig. 2D). To visually demonstrate the genetic impact of this process, we constructed a detailed map of *indica* introgressions and found that *indica* introgressions were present on all 12 chromosomes of NEC cultivars (Fig. 4B). The percentage of *indica* introgression was low in the cultivars from Japan but significantly higher in NEC cultivars (Fig. 4A). In NEC, cultivars from LN showed the highest level of *indica* introgressions, with an average of 80.1 Mb per cultivar. In HLJ province, the average content of *indica* introgressions in a cultivar increased significantly over time, from 8.2 Mb before 1980 to 76.6 Mb after 2010, which reflects its preference in breeding (Fig. 4A and Supplementary Fig. S5).

In the breeding process, *indica* introgressions with beneficial effect are more likely to be inherited. Accordingly, we focused on the introgression segments with high frequency in NEC (Fig. 4B and Supplementary Table S8A). A large *indica* introgression region with the highest frequency is located on chromosome 12, which introduced the resistant allele of one or both of the two adjacent rice blast resistance loci, *Pi-ta* [39] and *Ptr* [40], which significantly improved blast resistance in *japonica* rice [41]. Other highly frequent introgressions on chromosomes 2, 3, 5 and 6 may also introduce beneficial traits from *indica* (Fig. 4B). In HLJ, some introgressions showed increasing frequency since the 1980s. These included the one harboring *Pi-ta* and *Ptr* on chromosome 12, which was increased from 6% before 1980 to 37% after 2010, and the one harboring hybrid male sterility locus *Sc-I* [42] on chromosome 3, which was increased from 0% before 1980 to 39% after 2010 (Supplementary Fig. S5).

**Figure 5.**
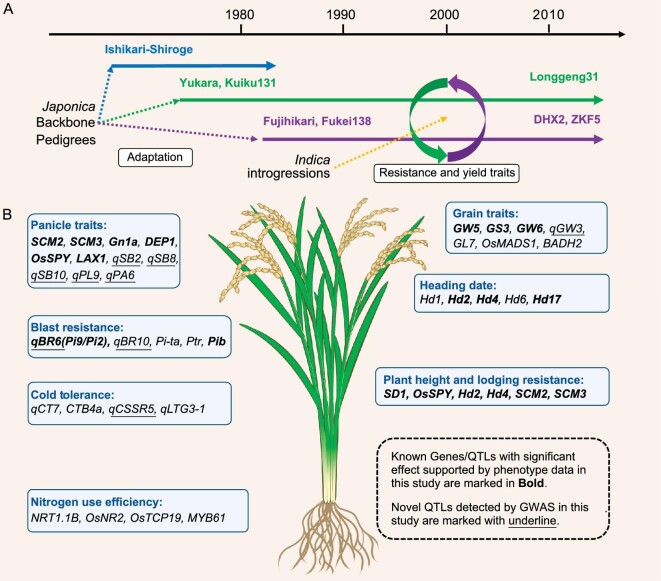
Inferred contribution of pedigrees, *indica* introgressions and applicable QTLs in NEC rice breeding. (A) An inferred model of rice breeding history in Heilongjiang province of NEC. Three major *japonica* subgroups with representative backbone cultivars and introgressions from *indica* rice that collectively contributed to genetic and phenotypic diversity. (B) Applicable QTLs collected from multiple methods, regulating multiple agronomically important traits. Known genes/QTLs with significant effect supported by phenotype data in this study are marked in bold. Novel QTLs detected by using GWAS in this study are marked with underline.

In K3G2 and K3G3, the proportion of *indica* introgression in cultivars showed significantly positive correlation (Supplementary Table S8B) with panicle traits, including panicle length, primary and/or secondary branching number per panicle and grain number per panicle. Using GWAS, we detected QTLs for several yield traits that were associated with *indica* introgressions. These include several QTLs on which *indica*-derived alleles positively contribute to grain width (Fig. 4C), panicle length (Fig. 4D) and branching number (Fig. 4E and F), consistently with the correlation analysis and previous studies [43]. The effects of three introgression-related QTLs on Chr2, Chr8 and Chr10 for panicle traits, named as *qSB2, qSB8* and *qSB10*, respectively, were further verified by using chromosome segment substitution lines (CSSLs) of Koshihikari (temperate-*japonica*) background carrying IR64 (*indica*) introgressions. Introgression of *indica* segments carrying *qSB2, qSB8* and *qSB10*, respectively, increased the secondary branching number per panicle by 100% and grain number per panicle by 50% (Fig. 4G and H, Supplementary Fig. S6 and Supplementary Table S9). These results proved the substantial contribution of *indica* introgressions to the improvement of yield traits in NEC rice breeding (Fig. 5A).

### Decoding past breeding practice has guiding value in BbD

Based on our above analyses, to achieve a coordinated improvement in grain yield, quality and stress tolerance in NEC rice breeding, the following principle or trend will be expected. (i) Given that the group K3G2 that dominates HLJ is being replaced by K3G3 over time (Fig. 2E), it may be better to choose elite varieties of K3G3 as the starting platform of design breeding. (ii) Increase yield or rice blast resistance by pyramiding more *indica*-derived segments or QTLs including *SCM2, qSB2, qSB8, qSB10, Ptr, Pi-ta* and unknown loci to be identified. (iii) Improve grain quality to cater to the demand of consumers by introducing multiple grain shape QTLs, such as the weak/null allele of *GW5, GS3* or other alleles that increase grain length. (iv) Use other gene alleles in our list (Fig. 5B) rationally and flexibly to breed cultivars that can better adapt to the environment and cultivation conditions in NEC, such as using the *qCSSR5* locus to improve cold tolerance, using the *SCM3* and *OsSPY* linkage to tackle lodging at the late maturity stage caused by typhoons that more frequently occurred in NEC in recent years [44], etc.

**Figure 6.**
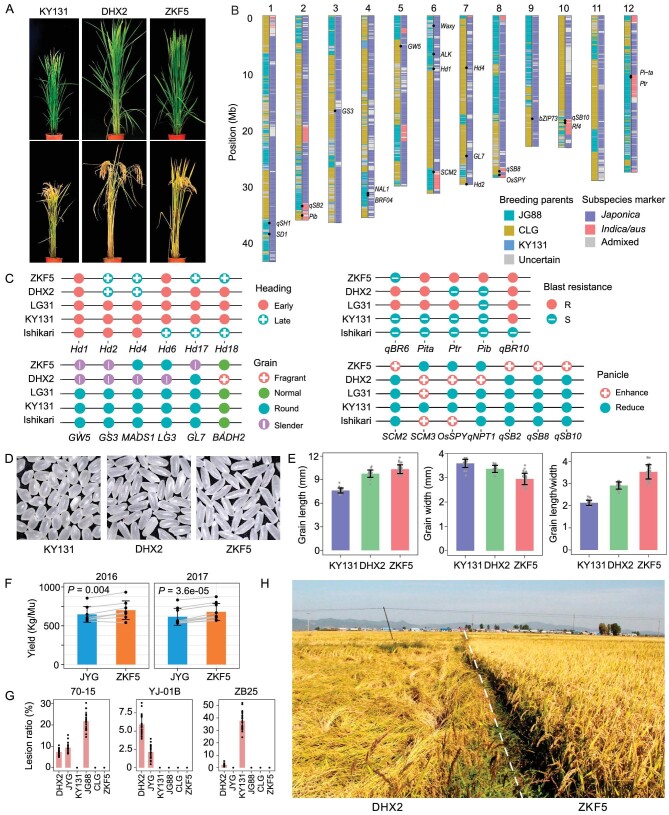
Genomic decoding of breeding history of an elite *japonica* cultivar, Zhongkefa5. (A) Plant phenotype of three elite cultivars: KY131, Daohuaxiang2 (DHX2) and Zhongkefa5 (ZKF5) at heading and mature stage. (B) Genome contribution of three breeding parents of ZKF5 and distribution of *indica* introgressions; favorable QTLs and breeding targets are marked on the chromosomes. (C) Allele combination of important QTLs in elite cultivars and early cultivar Ishikari-Shiroge (Ishikari) for comparison. For blast resistance QTLs, R for resistant allele and S for susceptible allele. (D) and (E) Grain phenotype of KY131, DHX2 and ZKF5. (F) Yield of ZKF5 compared to the control cultivar Jiyugeng (JYG) in nine experimental sites of Northern China in 2016 and 2017 (Supplementary Table S11); *P*-values are for Student's *t*-test. (G) Leaf lesion area ratio of ZKF5 and five other cultivars inoculated with different *Magnaporthe oryzae* isolates. (H) Field phenotype showing the lodging resistance performance of ZKF5 compared to DHX2.

In the practice of rice production, since 1990, KY131 from K3G2 has been in an absolutely dominant position in the NEC rice planting area [45]. From 2013 to the present, KY131 was replaced by Longgeng31 (LG31) with enhanced blast resistance from the same subgroup [46]. The success of KY131 and LG31 largely benefits from the recognition of rice growers for their stable yield, early maturity, cold tolerance, lodging resistance and acceptable quality [45,46]. These features may be attributed to the pyramid of a number of excellent natural alleles as we listed based on genome analyses (Fig. 6C). From the perspective of consumers, high-quality rice has much more commercial value and the most typical example is Daohuaxiang2 (DHX2, Fig. 6A), which has all indicators at the highest level of rice quality standards fixed by the Ministry of Agriculture and Rural Affairs of China [47] and is always in shortage. Therefore, breeding rice cultivars with high stable yield and high quality is still the goal and top priority.

Recently, Jiayang Li's team and their collaborators bred a new elite variety with both high yield and good quality, namely Zhongkefa5 (ZKF5, Fig. 6A). After decoding the breeding history of ZKF5 from a genomic perspective (Fig. 6B and C, and Supplementary Table S10), we demonstrated that it essentially provided practical validation for the breeding trend sorted out based on our analysis. ZKF5 was selected from a multi-parental cross population using Jigeng88 (JG88), an elite cultivar from K3G3, as the backbone cultivar to hybridize successively with KY131 from K3G2, and Nanfangchangligeng (CLG), a *japonica* cultivar from South China rice planting areas carrying a multi-copy *GL7* allele that increases grain length but has not yet been detected in NEC varieties, *Pib* allele that confers rice blast resistance and several *indica* introgression regions that are expected to improve yield (Supplementary Table S5 and Supplementary Fig. S7). In terms of appearance quality, genomic characterization confirmed that ZKF5 has a genotype combination of *GL7-*multi-copy, *gs3* and *GW5*, resulting in long/slender grains comparable to DHX2 (Fig. 6D and E) that has a genotype combination of *gs3, GW5, osmads1* [48] and *GW6* (Fig. 6C and Supplementary Fig. S8). For yield improvement, ZKF5 harbors *qSB2, qSB8, qSB10* and *SCM2* (Fig. 6B and C, and Supplementary Fig. S8) and reached an average yield of 700.48 and 678.50 kilogram per mu (1 mu = 1/15 hectare) in 2 years of rice regional trials, which is about 8.36% and 10.22% higher than those of Jiyugeng (JYG), the officially designated control cultivar for regional trials, respectively (Fig. 6F and Supplementary Table S11). Moreover, ZKF5 carries the blast resistance alleles of *Pib, qBR10, Pi-ta* and *Ptr* (Fig. 6B and C, and Supplementary Fig. S8), making it much more resistant to rice blast than JYG (Fig. 6G and Supplementary Fig. S9). Notably, compared with DHX2, ZKF5 showed excellent lodging resistance under natural field conditions (Fig. 6H), supporting the successful selection of appropriate plant height and culm strength.

## DISCUSSION

In this study, to accurately assess the trend of rice breeding in the new period, we took systematic analyses of the chronological characteristics of the past breeding practices in NEC from a genomic perspective. We genotyped 111 known QTLs and novel GWAS-QTLs in our rice collection (Supplementary Tables S5 and S7). Among them, some QTLs contribute to the basic adaptation traits in NEC, such as *Hd1* for proper heading date [20], while others were alterable for designing specific cultivar for a specific need [17]. Some QTLs that have been widely used in NEC and with promising effect deserve further concern, such as *Hd2, Hd4, SCM3, SCM2, DEP1* and *qBR6*. Some QTLs that may introduce unfavorable traits should be avoided in breeding, such as the blast susceptible allele of *qBR10*. Also, some QTLs for disease resistance that may have made a historical contribution to resistance breeding in NEC, such as *Pi-ta* and *Ptr* [41], now face the threat of lost resistance due to long-term application, which strongly reminds rice breeders that introducing new resistance loci is needed. We noticed that many reported QTLs were not detected by using GWAS, which may be due to the influence of population structure, complex interaction between loci and environment effects or limitations of the algorithm, and new methods for GWAS may be able to get more results. Estimating the additive and dominance effects of QTL, as well as the controlling polygenic backgrounds, should be a promising approach to improve statistical power and avoid confounding effects. Recently, a comprehensive GWAS method for dissecting quantitative traits, 3VmrMLM, was developed and released in software package IIIVmrMLM [49]. Adopting this method for our data confirmed some of the QTLs we found by using EMMAX and also many other novel QTLs (Supplementary Table S12) that may be worth further verification. This suggests that reanalysing old data with new methods could yield more valuable information.

Although NEC cultivars are dominated by *japonica* rice background, introgressions from *indica* sub-species have also contributed to the breeding in NEC, as documented by previous studies [17,38]. Based on GWAS analysis, we confirmed that some chromosome segments that were introduced into *japonica* cultivars from inter-sub-species crossings harbor major QTLs contributing to resistance and yield traits, further highlighting the importance of broadening genetic diversity during NEC rice breeding. Although more introgression regions of *indica* sub-species are beyond the range of QTLs detected by our GWAS, it is more likely that these regions may be related to some important traits that we did not collect. Consistently with this speculation, by analysing the chronological distribution trend of these fragments, we found that some introgression chromosome regions appeared with increasing frequency in NEC rice cultivars over time (Supplementary Fig. S5), suggesting that these regions may be preserved due to an unknown role in NEC rice improvement. These regions deserve special attention in functional analysis and rice breeding in the future. ZKF5 now serves as the main cultivar in HLJ and JL provinces with its high yield, good quality and stress-tolerant features. Our analysis has decoded the genomic history of its successful breeding and, moreover, based on our analysis, ZKF5 may be further improved by utilizing beneficial genes such as *Hd2, Hd4, Hd17* and *Hd18* for earlier heading and *SCM3, OsSPY, qNPT1* and nitrogen use efficiency-related alleles for higher yield, pinpointing the further breeding path. As a longer-term breeding goal in NEC, it is necessary to analyse the excellent gene alleles harbored in important loci such as *qCSSR5, qSB2, qSB8* and *qSB10* and in *indica* rice introgression fragments selected with high frequency, so as to achieve more precise and more comprehensive trait improvements, which are expected to further validate the guiding value of our analyses on BbD in practice. In addition, the construction of CSSLs to clarify the potential utilization value of other introgression fragments from different rice regions or sub-species, which have not yet been integrated into NEC cultivars, will also help to provide more diverse genetic resources for the future improvement of rice varieties in NEC.

It is worth noting that low genetic diversity is one of the biggest bottlenecks in rice breeding in NEC, which is bound to be aggravated in the long run if it is limited to select backbone parents in a specific group for improvement. In addition, BbD of rice varieties including ZKF5 at this stage is limited to the design and pyramiding of a handful of gene loci with large effects. The design and pyramiding of large-scale important agronomic trait loci, especially those with minor effects, will depend on the application of breeding strategies including genomic selection [4,50,51]. That is, BbD of backbone parents in NEC using genomic selection by referencing the core trait loci with chronological features as we provided (Figs 3–5) in the future may be a rational way to contribute to the innovation and diversification of breeding resources in this region.

Overall, our study analysed the chronological characteristics of rice breeding practice in NEC from the perspective of functional genomics and dissected basic information that was absent in the past but is very important for future BbD. Similar strategies can be applied to other rice or crop breeding regions, which may accelerate BbD of different crops worldwide.

## MATERIAL AND METHODS

### Plant materials and phenotype evaluation

The rice population was collected from multiple rice institutes in Heilongjiang, Jilin, Liaoning, Korea and Japan. Most of the agronomic trait phenotyping was performed in Harbin (45°N, China) in 2014. Cold tolerance measurements were performed in the lab. Field blast measurements were performed in Shangzhi (44°N, China) in 2010 and 2011 according to the grading criteria formulated by the International Rice Research Institute [52]. CSSLs derived from a cross between Koshihikari and IR64 were kindly provided by Nagata *et al.* (https://www.rgrc.dna.affrc.go.jp/index.html.en) [53]. Details are provided in the Supplementary data.

### DNA sequencing and data processing

DNA libraries (400–500 bp) were prepared and sequenced by using a Hiseq2000 genome analyser (Illumina, San Diego, CA). Sequencing data were aligned to the Nipponbare reference genome (version 7.0) [54] using Burrows-Wheeler Alignment (BWA) Tool [55]. Data analysis were performed using samtools [56] and GATK [57] (version 2.7.2). Details are provided in the Supplementary data and Supplementary Fig. S10.

### Population structure inference

We performed population structure inference using Admixture [58]. Principle component analysis was performed using smartpca [59]. A neighbor-joining tree was constructed by using dna.dist and nj function in R package APE [60]. Details are provided in the Supplementary data.

### Genome-wide subpopulation ancestry inference and inter-subpopulation introgression inference

The procedure of subpopulation ancestry inference was described previously, based on 3K-SNP and 3K-HAP data sets [17]. We further used *f*_dM_, a statistic related to Patterson's *D* implemented in Dsuite software [61], to test whether these putative introgression events overlap with Admixture events between subpopulations. Details are provided in the Supplementary data.

### Measuring nucleotide diversity and differentiation

Fixation index (*F*_ST_) and nucleotide diversity (π) were calculated using VCFtools (version 0.1.15) [62]. Details are provided in the Supplementary data.

### Genotyping of known QTLs

Known QTLs (Supplementary Table S5) were collected and genotyped as described [63,64]. Gene copy number variation was detected as described [65]. Allele effects were defined according to their publications as follows: WT for functional wild-type; High for higher protein function or expression level; Low for lower protein function or expression level; LoF for loss-of-function due to broken protein or loss of gene sequence. Details are provided in the Supplementary data.

### Genome-wide association study

GWAS were performed using EMMAX with the mixed linear model (MLM) [66]. We also performed GWAS with a new method, IIIVmrMLM [49], with default parameters. Details are provided in the Supplementary data.

### Breeding procedure of ZKF5

CLG as the male parent was first crossed with KY131, and the resulting F_1_ plants as the female parent was further crossed with JG88 to generate a segregating population, which was bred from F_2_ generation to F_9_ generation, so as to obtain stable lines. In the meantime, each generation was screened through agronomic preference evaluation in the field and molecular marker-assisted selection (Supplementary Table S10 and Supplementary Fig. S8) in the lab to confirm the genotype combination of yield, disease resistance and grain shape.

### DATA AVAILABILITY

Raw FASTQ reads for all rice accessions whose genomes were sequenced for this study have been deposited in the SRA under Bioproject accession number PRJNA844290.

## Supplementary Material

nwad029_Supplemental_Files
