# Supplementary material for: Genomic decoding of breeding history to guide breeding-by-design in rice
Source: Natl Sci Rev. 2023 Feb 9;10(5):nwad029. doi: 10.1093/nsr/nwad029 (PMC10089590; doi:10.1093/nsr/nwad029)
Supplement: nwad029_Supplemental_Files [file nwad029_supplemental_files.zip › Supplemental_Figures.docx]

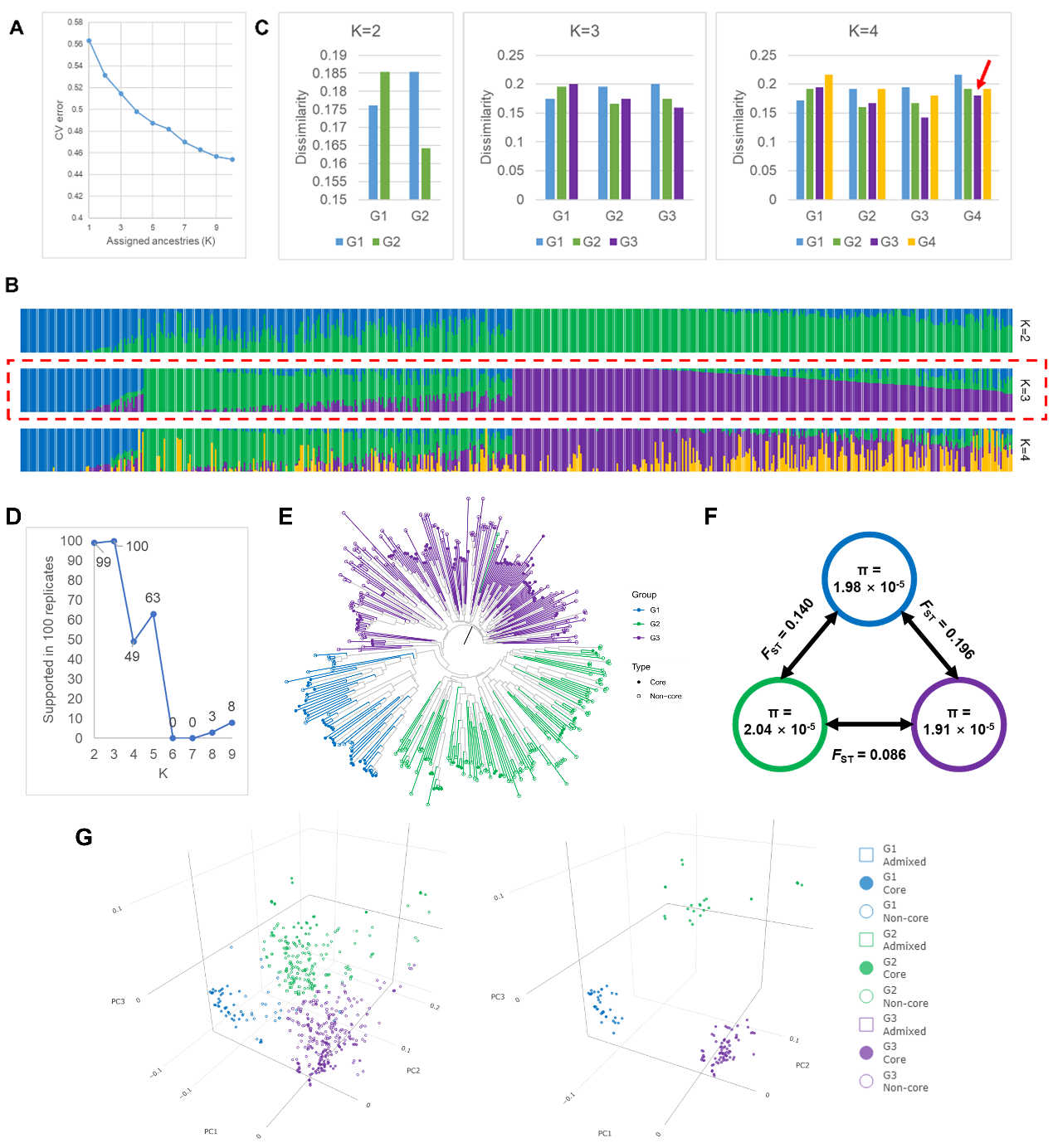


Fig. S1 Population structure inference of NEC rice cultivars.

(A) Cross validation error of inference assigning different number of ancestries. (B) Tabulated results of population structure inference. (C) Pair-wise dissimilarity of samples in each inferred subgroups when assigning 2, 3, and 4 ancestries. (D) Times of replicates supported for each K in 100 replicates of Admixture analysis with random subsets of markers. (E) Neighbor-join tree of samples from three inferred groups. (F) Genome-wide average π values in each subgroup and *F*_ST_ values between each two subgroups. (G) 3D-PCA plot of all and core cultivars.


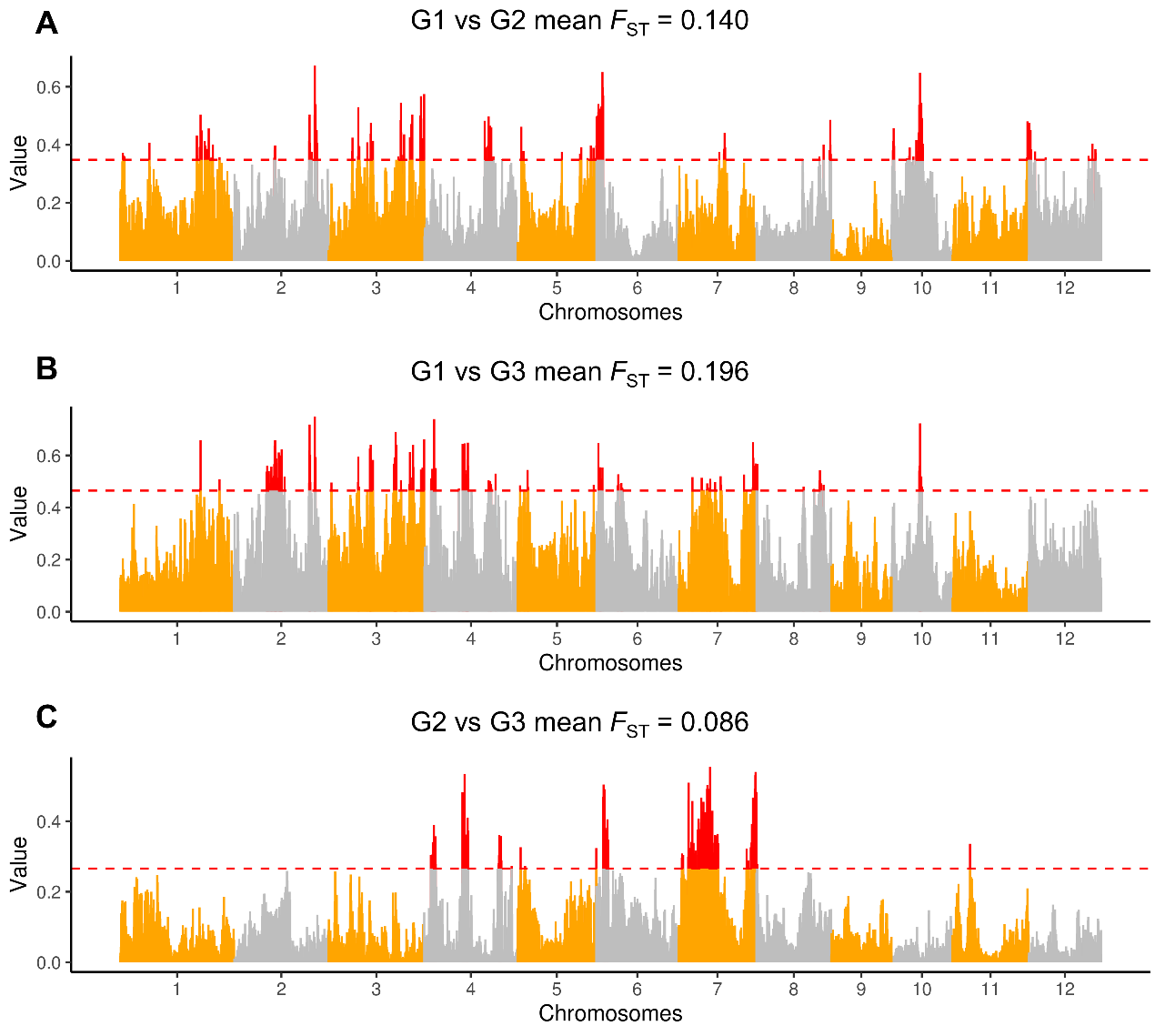


Fig. S2 Distribution of *F*_ST_ value between the three subgroups.

Weighted *F*_ST_ values by 100-kb window between: (A) G1 and G2, (B) G1 and G3, and (C) G2 and G3. Top 5% windows in each comparison were marked in red. The red dashed line marked the threshold of top 5% windows in each comparison.


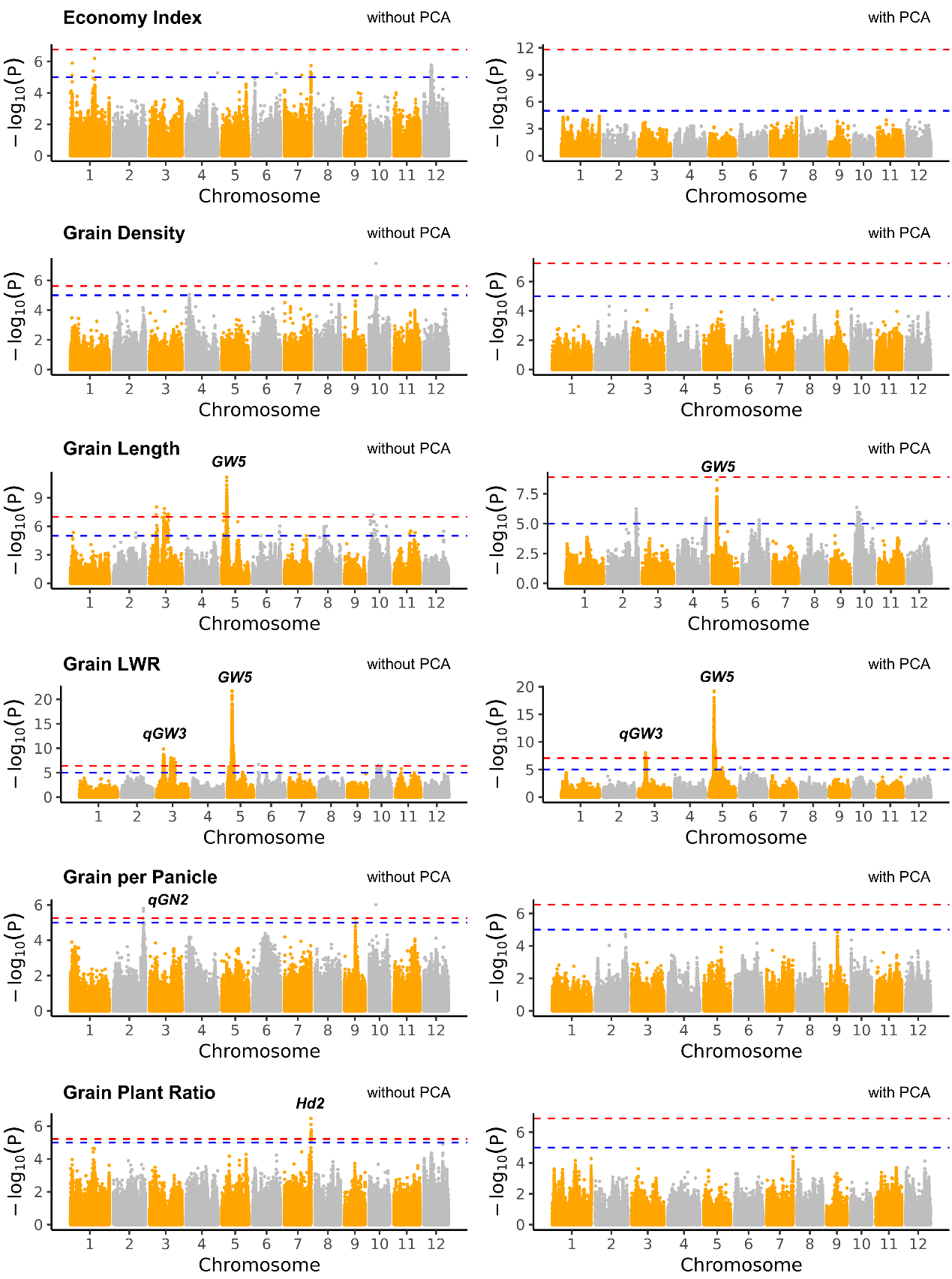
**Fig. S3. Manhattan plots of GWAS of 22 traits by EMMAX.**

In each plot, the red dashed line indicates the threshold of -log_10_ *P*-value obtained from permutation test for each trait (Table S7C). The blue dashed line indicates an empirical threshold of 5. GWAS were performed using EMMAX MLM with and without top 3 PCs which separated the three subgroups as covariates.


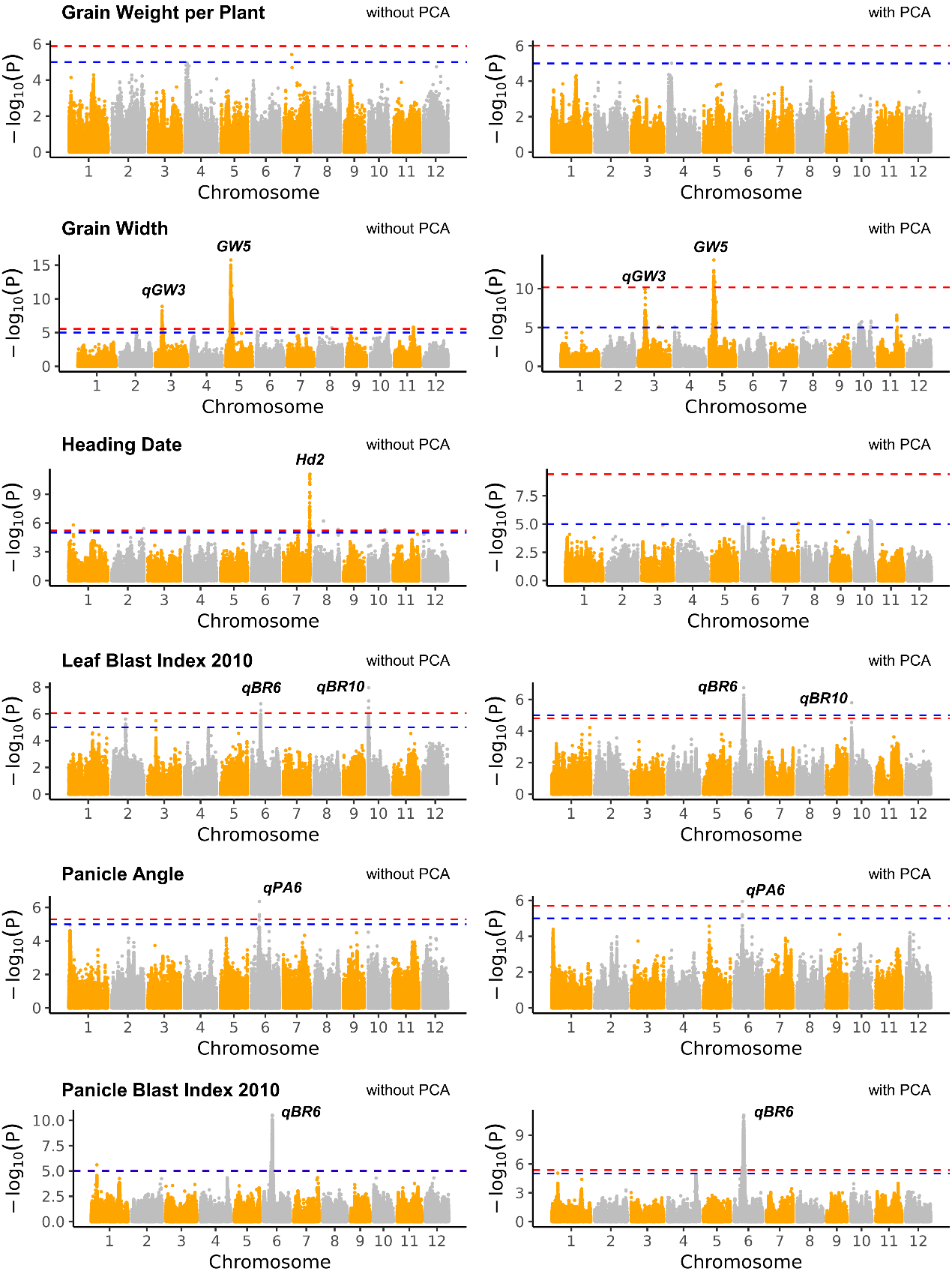
Fig. S3. (Continued)


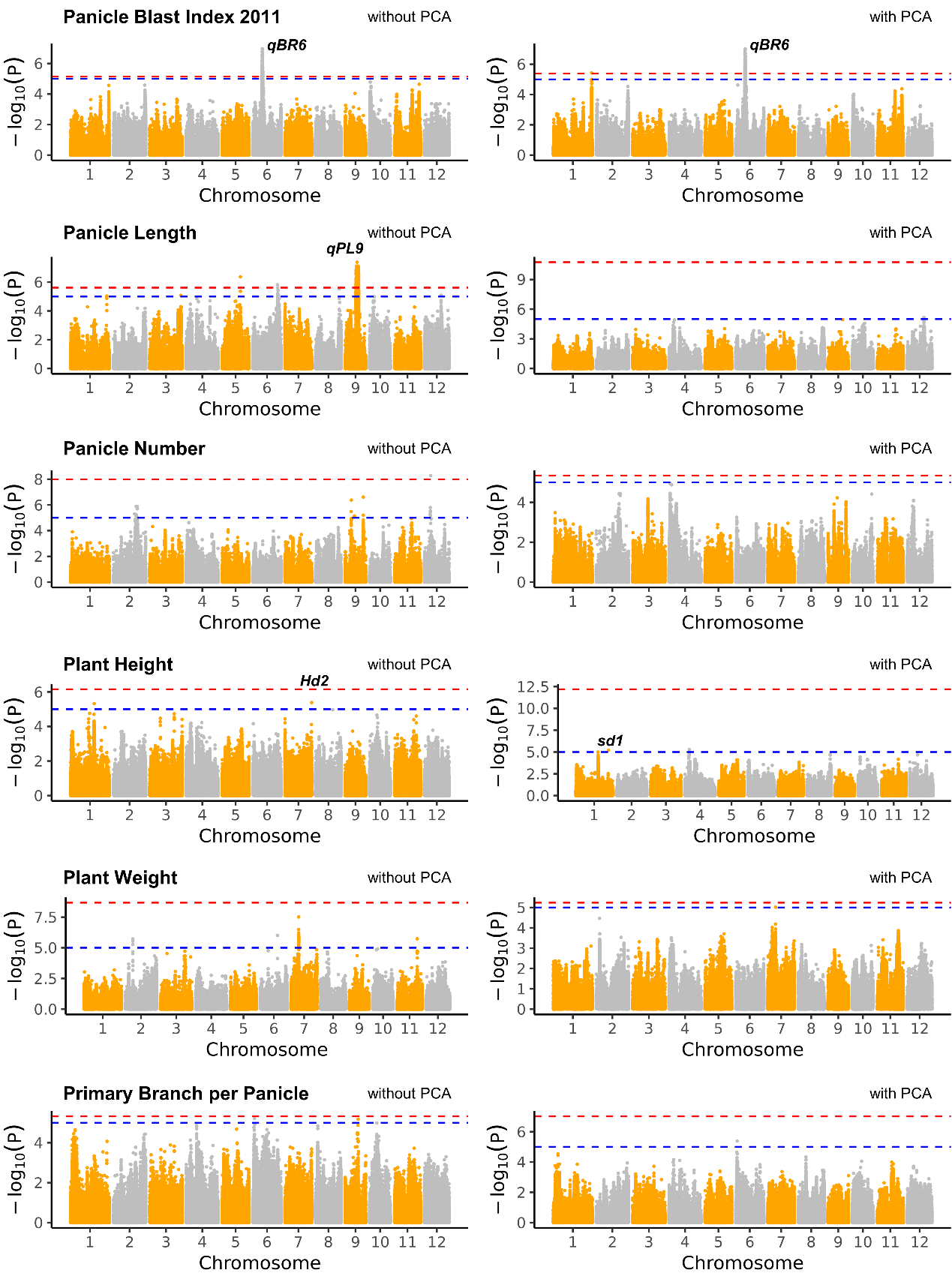
Fig. S3. (Continued)


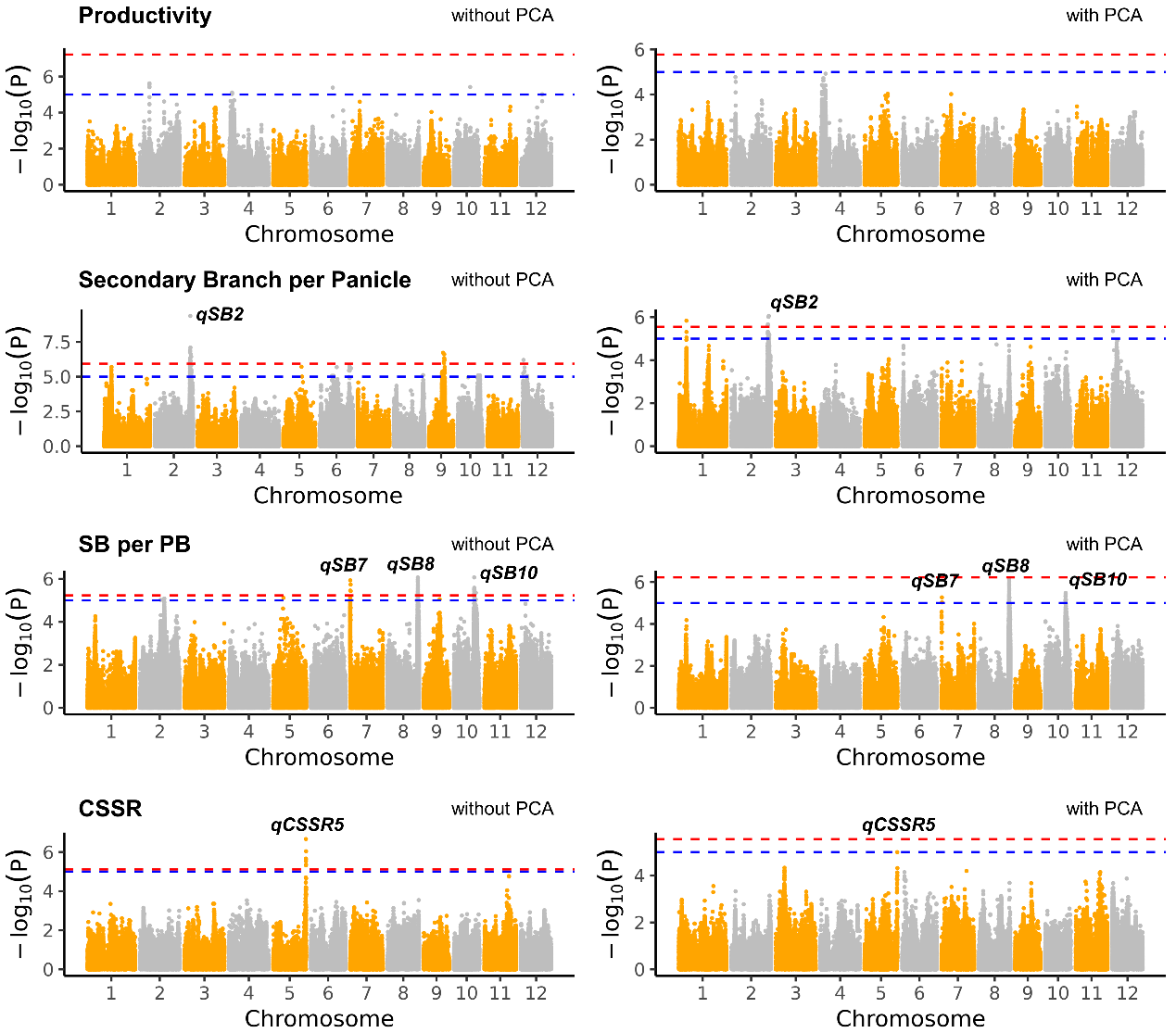
Fig. S3. (Continued)


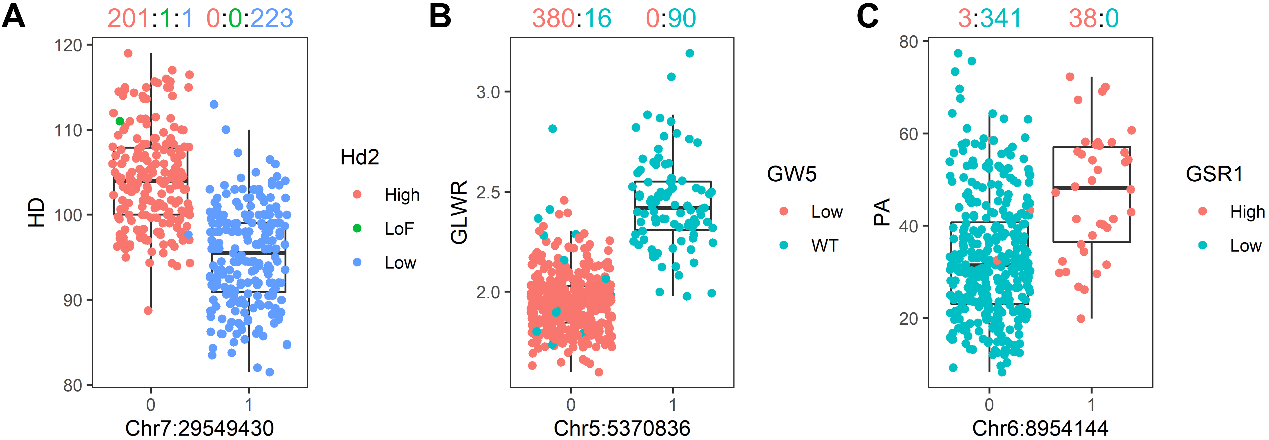


**Fig. S4. Correspondence of GWAS peak SNPs and candidate genes with reported causal variants.**

The details of reported functional allele are provided in Table S5A. HD for heading date; GLWR for grain length and width ratio; PA for panicle angle.


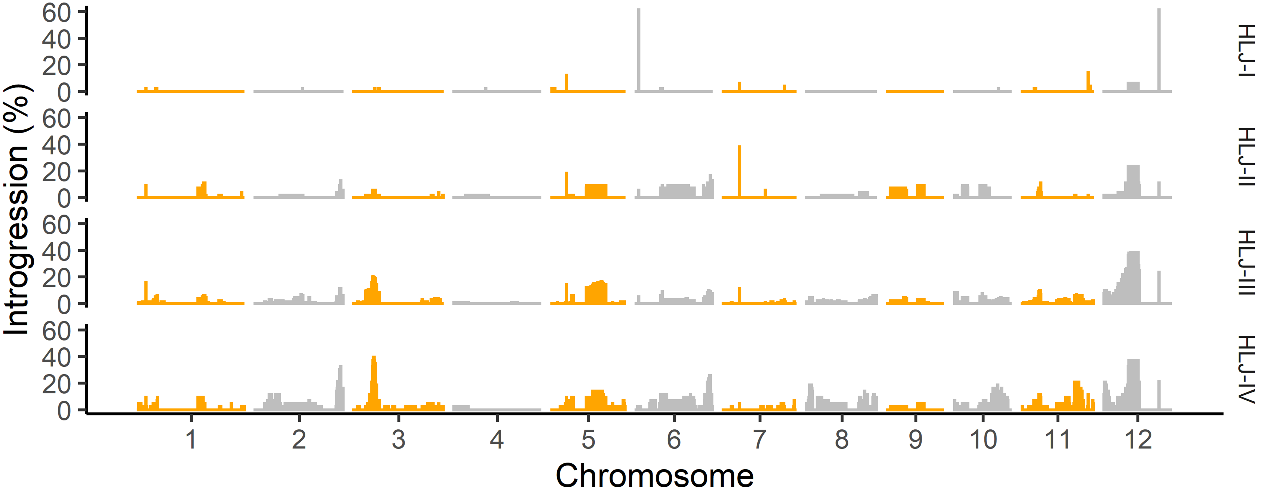


Fig. S5 Distribution of *indica* introgressions in four breeding eras in HLJ province.

HLJ-I for cultivars before 1980; HLJ-II for cultivars between 1980 and 2000; HLJ-III for cultivars between 2000-2010; HLJ-IV for cultivars after 2010.


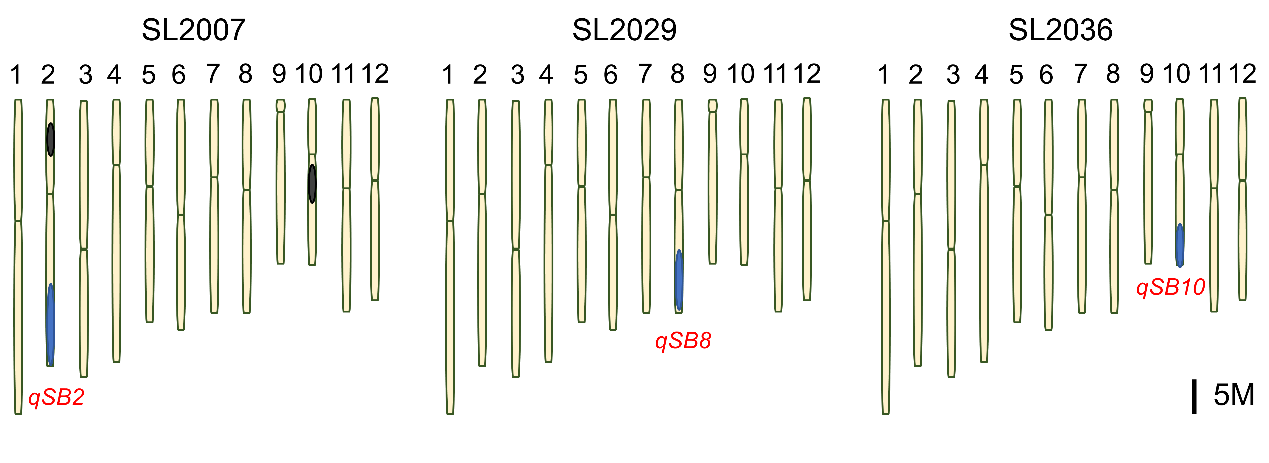


Fig. S6 Distribution of IR64-derived segments in the three CSSLs. Data from Nagata, K. et al.

CSSL plant materials from “Advanced backcross QTL analysis reveals complicated genetic control of rice grain shape in a *japonica* x *indica* cross. Breed. Sci. 65, 308-318 (2015).”


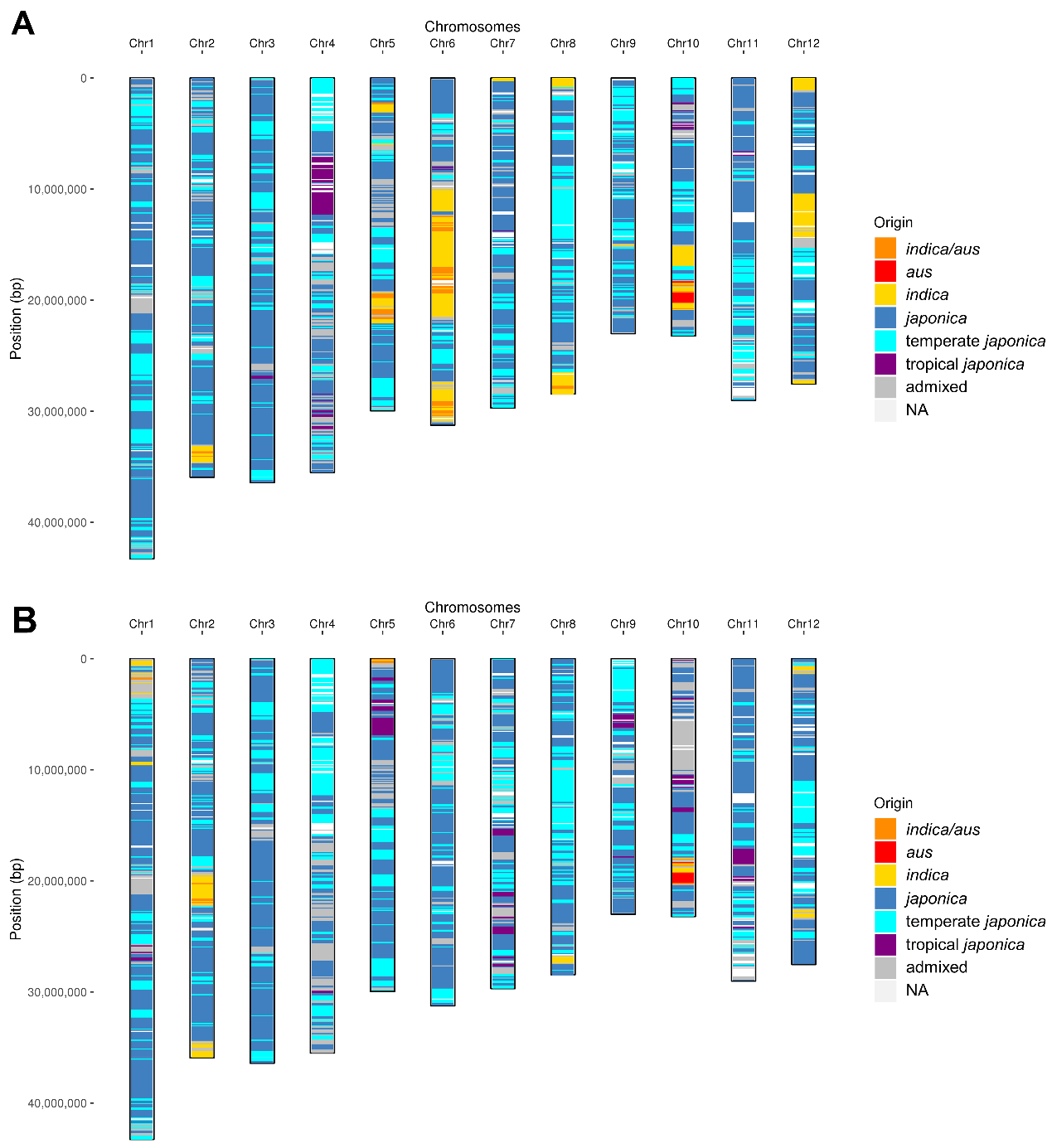


Fig. S7 Genome-wide subpopulation components of Jigeng88 (JG88) and Nanfangchangligeng (CLG).

Karyotype plot of inferred subpopulation component in 100-kb windows: (A) JG88; (B) CLG.


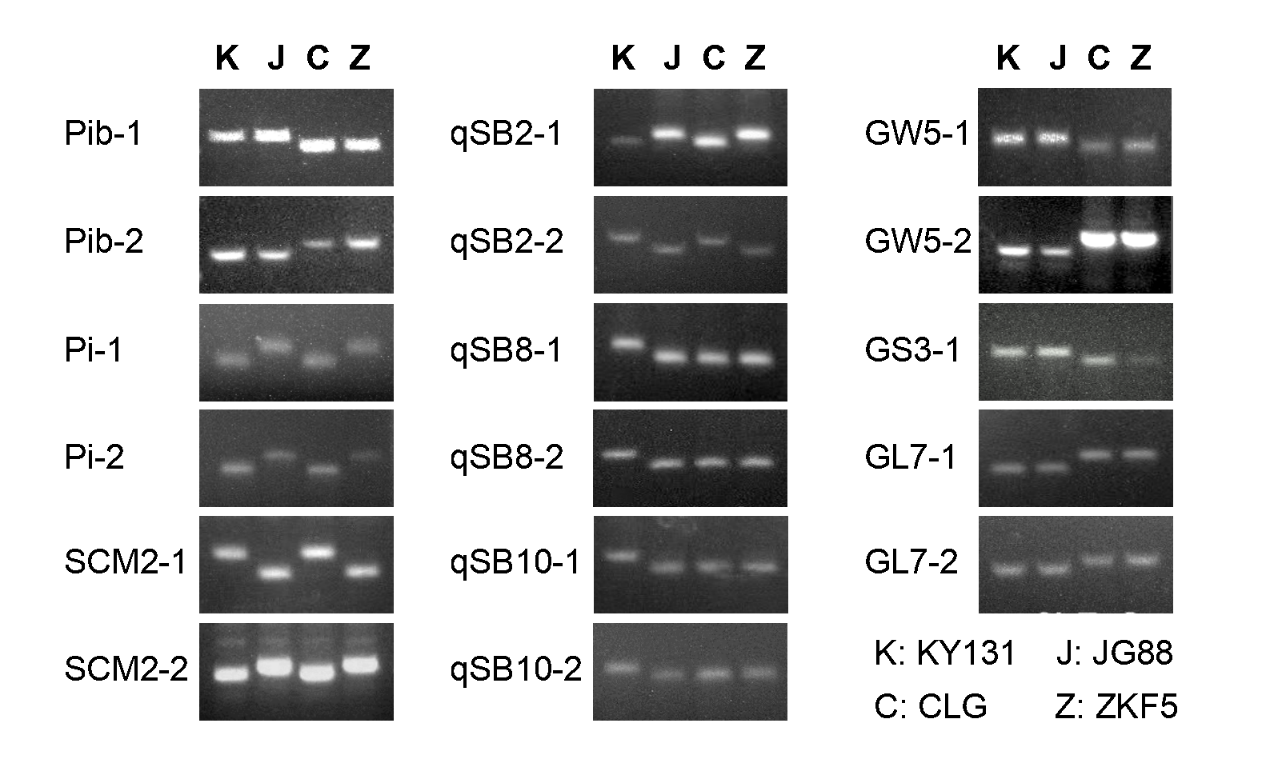


Fig. S8 Polymorphism of designed markers among KY131, JG88 and CLG

K, J, C and Z denote KY131, JG88, CLG and Zhongkefa5, respectively.


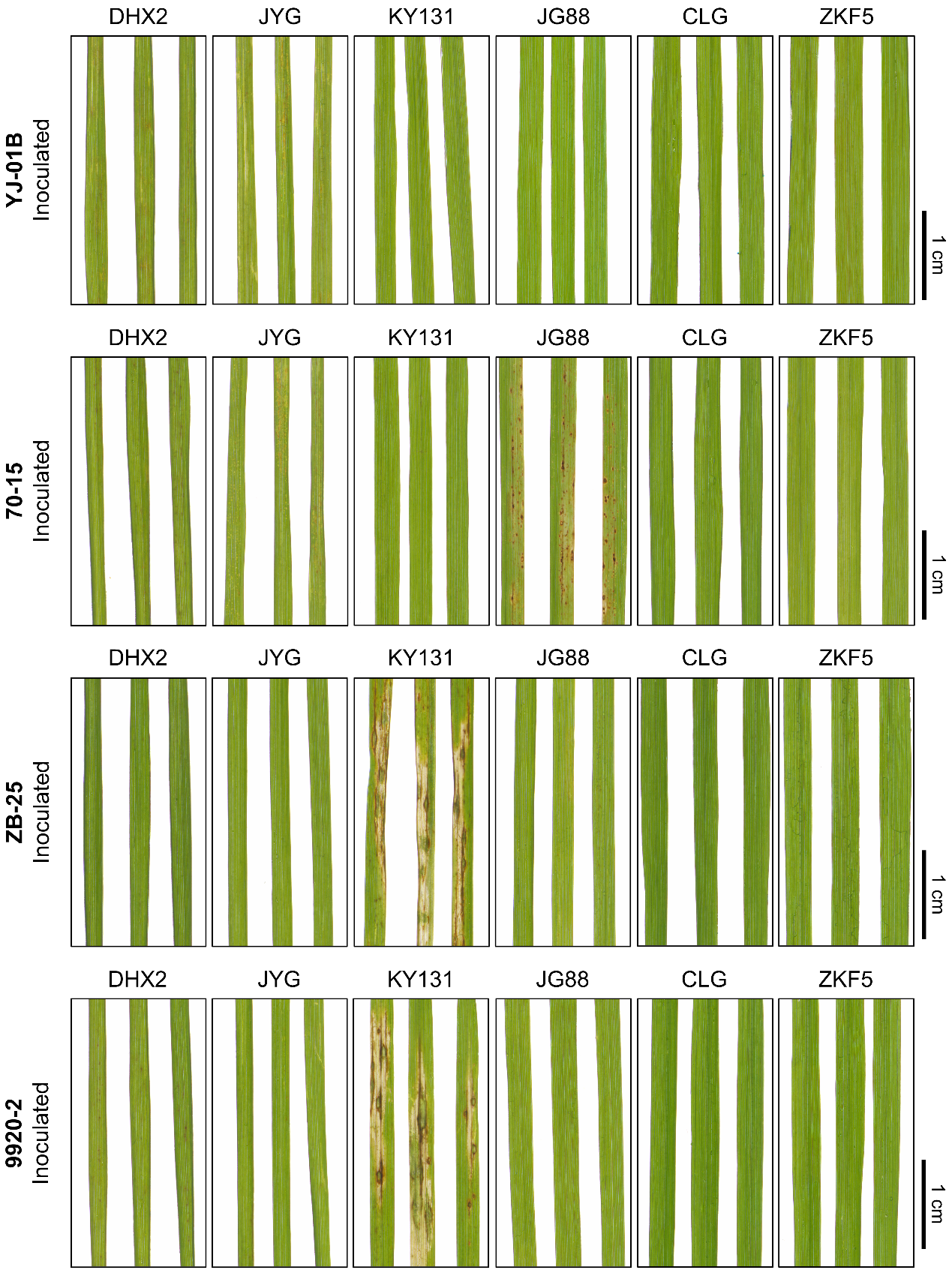
**Fig. S9 Representative leaves from *Magnaporthe oryzae* infection assays**

Leaves of Zhongkefa5 (ZKF5) and other 5 rice cultivars inoculated with 4 different *M. oryzae* isolates: (A) YJ-01B; (B) 70-15; (C) ZB-25.


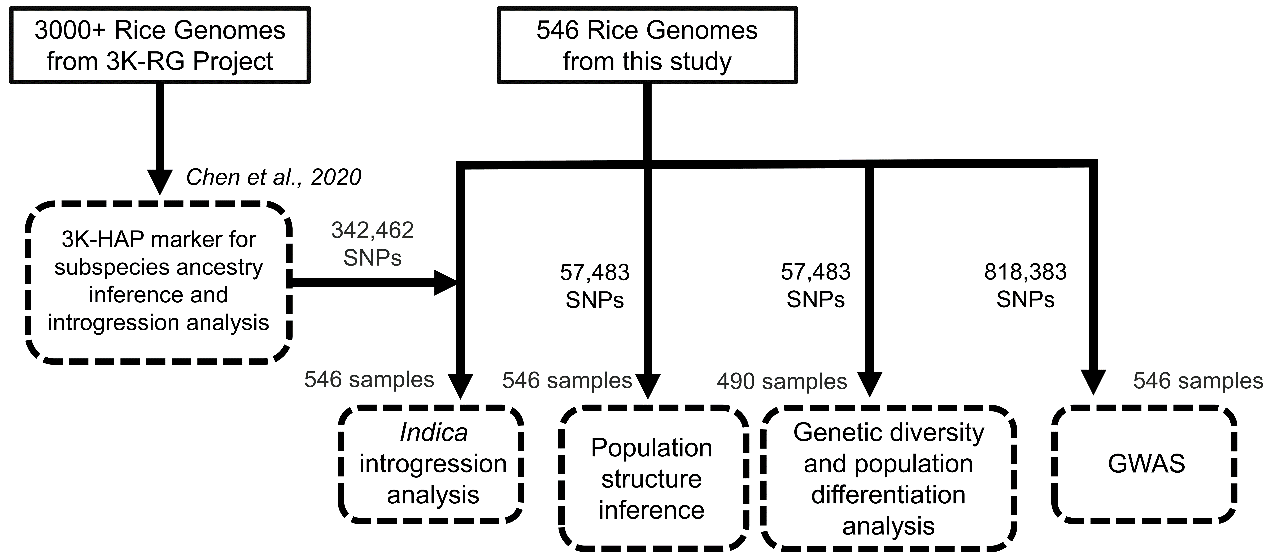


**Fig. S10. Summary of samples and markers used in this study.**


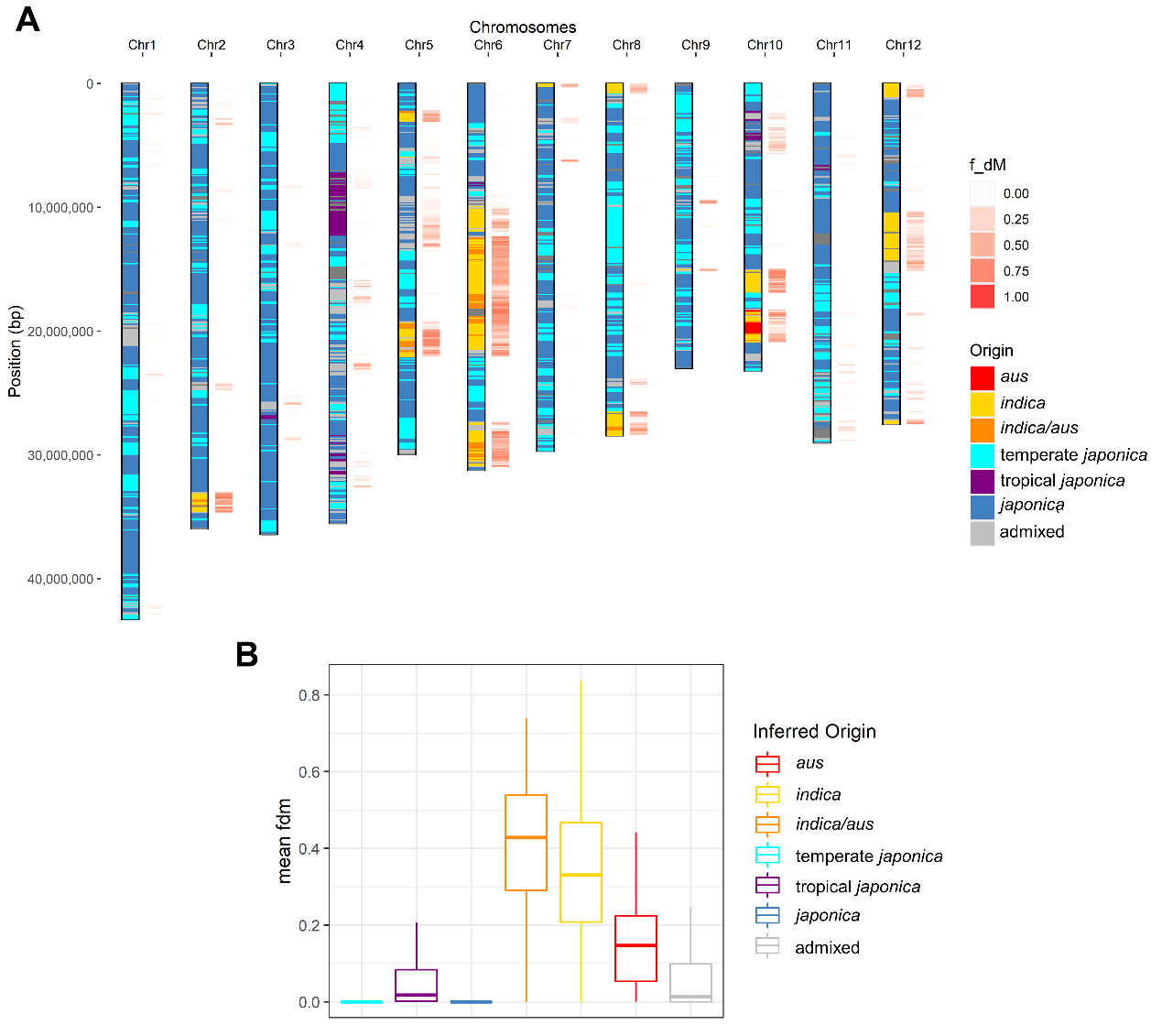


Fig. S11 Comparison of subpopulation inference by 3K-RG and inter-subspecies admixture inferred by Dsuite

(A) The left track of each chromosome shows genome-wide subpopulation inference of a temperate *japonica* cultivar, Jigeng88. The right track shows *japonica* x *indica* gene flows measured by *f*_dM_. (B) Mean *f*_dM_ value of all inferred windows in all sequenced temperate japonica samples, in which windows inferred as from *indica* showed significantly higher (*P* < 2.2 x 10^-16^) level of admixture with *indica* compared to windows inferred as *japonica* background.
