# Supplementary material for: Genomic decoding of breeding history to guide breeding-by-design in rice
Source: Natl Sci Rev. 2023 Feb 9;10(5):nwad029. doi: 10.1093/nsr/nwad029 (PMC10089590; doi:10.1093/nsr/nwad029)
Supplement: nwad029_Supplemental_Files [file nwad029_supplemental_files.zip › Supplemental_Methods.docx]

**MATERIAL AND METHODS**

**Plant materials and phenotype evaluation**

The rice population used in this study was collected from multiple rice institutes in Heilongjiang, Jilin, Liaoning, Korean, and Japan. All cultivars collected are grown on Yanjiagang Farm (45°N, Harbin, China) in 2014. Seeds were sown in seed beds in a greenhouse on early April and transplanted to the paddy field in the middle of May. Plants were grown followed the normal agricultural practices in the paddy field. Flowering time was recorded from sowing to the appearance of the first panicle. For agronomic trait phenotyping, after ripping, three individual plants for each cultivar were used for score. For plant and panicle traits, the phenotype data is the mean value of 5 plants for each cultivar. Productivity is the sum of the weight of grass and grain. Economy index is the grain weight per plant divided by productivity.

For cold tolerance measurement, each plant was grown in a cylindrical plastic pot. Approximately 10 d before booting, when the distance between the auricles of the flag leaf and the penultimate leaf of the tiller was within -5~0 cm (corresponding to the period between the meiotic stage to uninucleate pollen stage), tillers were tagged and pots were moved to a low-temperature greenhouse (15°C constant temperature, 14 h light/10 h dark photoperiod). After cold treatment for four days, treated pots were returned to the greenhouse under normal growth conditions until maturity. The seed-setting rate was calculated by the percentage of fully-filled grains per panicle relative to the total grain number per panicle. Five independent plants per cultivar were used for treatment.

For field blast measurement, the cultivars collected are grown in Shangzhi (44°N, Mudanjiang, China) in 2010 and 2011, where have been long used to select blast resistant varieties with high selection pressure of blast disease. Field rice blast resistance was evaluated according to the grading criteria formulated by the International Rice Research Institute (52). The evaluation results of each accession represent the index of the grading of three plots. Each plot contained three rows with 8 plants per row. The incidence of leaf blast was not obvious in 2011, we used the incidence data of leaf blast and panicle blast in 2010 and panicle blast in 2011 for further analysis. *Magnaporthe oryzae* isolates, ZB15, YJ-01B, and 70-15 were used for rice blast inoculation in the laboratory. All isolates were grown in a growth chamber at 25°C in a 12 h light / 12 h dark photoperiod for two weeks. Then, spores were collected via flooding of the fungal agar cultures with sterile water, and the spore concentration in the suspension was adjusted to 3×105 conidia/mL to spray-inoculate rice seedlings at the three-leaf stage (20 d after germination). The leaf was photographed and the lesion area was calculated at 7 days after inoculation.

Chromosome segment substitution lines (CSSLs) derived from a cross between a *japonica* cultivar Koshihikari and an *indica* cultivar IR64 were kindly provided by Nagata et al. (https://www.rgrc.dna.affrc.go.jp/index.html.en) (53).

**DNA sequencing and data processing**

We adopted a CTAB method to extract DNA. In brief, about 2 g of fresh seedling materials were grounded into fine powder in liquid nitrogen and thoroughly mixed with 2× CTAB DNA extraction buffer (100 mM Tris-HC1 [pH 8.0], 1.4 M NaCl, 20 mM EDTA [pH 8.0], 2% CTAB). After incubation at 28°C for 30 min, DNA was extracted with equal volume of chloroform and precipitated with 0.8 volume of isopropanol. Genomic DNA was washed twice with 99% ethanol before being dissolved in 100 μl of water. We determined the DNA concentration with Nanodrop 2000 (Thermo Scientific, Waltham, MA), and no less than 3 μg of DNA for each sample was used for sequencing library construction. DNA libraries (400–500 bp) were prepared and sequenced with a Hiseq2000 genome analyzer (Illumina, San Diego, CA) following manufacturer's instructions, with 90-bp paired end reads generated. The raw sequence data were further processed by removing adaptors and low-quality reads (more than 50% of bases have quality ≤5). The library preparation, genome sequencing, and raw data processing were conducted in BGI-Shenzhen, China. Sequencing data were aligned to Nipponbare reference genome (version 7.0)(54)using BWA(55) with MEM algorithm. Then the mapping results were filtered and sorted using samtools(56). Potential PCR duplications were removed using samtools rmdup function. Reads that may have been miss aligned due to putative insertions/deletions were marked and realigned using GATK (57) (version 2.7.2) RealignerTargetCreator and IndelRealigner functions. Nucleotide variants were detected using UnifiedGenotyper tool in GATK. Then the obtained variants were classified and tagged using VariantFiltration tools in GATK. Sample and marker numbers used in the following analysis are provided in Fig. S10.

**Population structure inference**

To perform population structure analysis, we used the following criteria to obtain a slim and stable collection of SNP markers. Firstly, based on the gene annotation of Nipponbare genome (MSU version 7.0), SNPs that fell within annotated transposable elements were removed. Secondly, considering the general homozygous nature of rice cultivars, SNPs that have a heterozygosity over 0.05 or over half of the minor homozygous allele frequency were removed. Finally, SNPs that fell within the same 100-kb window with highly correlated genotype distribution (*r*^2^ > 0.81) were merged and those with lower missing rates were kept. In sum, we obtained a collection of 57,483 bi-allelic SNPs for the following population genetics analysis. We performed population structure inference using ADMIXTURE (58). To further confirm the reliability of the grouping, we created 100 subsets of the SNPs by randomly picking 1 SNP in each 100-kb window and performed ADMIXTURE analysis with each subset. For each number of assigned ancestries (K), a subset with all inferred groups supporting groups inferred by the total set (with intersection between the two inferred groups accounted for more than 75% of each other) was defined as consistent with the total set. The assignment of a cultivar to a group that was supported in at least 75% of the replications was considered reliable. Principle component analysis was performed with the same dataset using smartpca (59). Neighbor-Joining tree was constructed using dna.dist and nj function in R package APE (60).

In each subgroup defined by Admixture, cultivars that were reliably assigned to the subgroup with ancestry score over 0.99 were defined as ‘core’ cultivars, those with ancestry score between 0.66 and 0.99 were defined as ‘non-core’ cultivars and the rest as ‘admixed’.

**Genome-wide subpopulation ancestry inference and inter-subpopulation introgression inference**

The procedure of subpopulation ancestry inference was described previously, based on 3K-SNP and 3K-HAP datasets (17). We described the major steps as follow: firstly, all sequenced cultivars were genotyped on the on the 3K-SNP sites with GATK UnifiedGenotyper tool, adopting a “--output_mode EMIT_ALL_SITES” parameter. Then haplotypes were constructed by joining the SNP genotypes in each window. The NAF-scores of each haplotype were obtained by matching with 3K-HAP and the average value for each 100-kb window was taken. Subspecies or subpopulation ancestry of each window was inferred according to the NAF-score. For a sample that is assigned to a certain subpopulation, a window that possesses a differed subspecies or subpopulation origin indicates a putative alien introgression. We further used *f*_dM_, a statistic related to Patterson's *D* implemented in Dsuite software (61), to test whether these putative introgression events overlap with admixture events between subpopulations. Ten *Oryza glumaepatula* accessions (NCBI Bioproject ID: PRJDB4703) were used as outgroups (O), temperate-japonica accessions in 3K-RG as P1, and indica subspecies as P3. Genome-wide calculation of *f*_dM_ was performed using a window size of 20 SNPs and step size of 10 SNPs (Fig. S11).

**Measuring nucleotide diversity and differentiation**

Fixation index (*F*_ST_) was used to measure the genetic differentiation between different subgroups and nucleotide diversity (π) were used to measure genetic diversity from the same 57,483 bi-allelic SNPs as population structure analysis. We calculated *F*_ST_ and π for each 100-kb window using VCFtools (version 0.1.15) (62). For subgroups defined by ADMIXTURE, only cultivars with reliable assignment were used for the analysis.

**Genotyping of known QTLs**

Known genes or QTLs with causal mutations or linkage makers reported in previous studies, which we called functionally verified natural variations (Table S5), were collected and genotyped as described (63, 64). SNPs and small indels were identified using GATK UnifiedGenotyper; large deletions relative to Nipponbare were identified on the basis of sequence coverage and large insertions genotyped based on the soft clips flanking the insertion sites. Gene copy number variation of GL7 was detected based on mapping depth of NGS data as described (65). For each functionally verified natural variation, allele effects were defined according to their publications as follow: WT for functional wild type; High for higher protein function or expression level; Low for lower protein function or expression level; LoF for loss-of-function due to broken protein or loss of gene sequence.

**Genome-wide association study**

SNP markers used for GWAS were obtained with similar procedure with population structure analysis, but without the pruning step. In total, 818,383 bi-allelic SNPs were used. GWAS were performed using EMMAX with MLM model (66). We performed PPMCC analysis using R basic function and found all traits showed weak correlation with the top 3 PCs. We performed GWAS with and without the top 3 PCs as co-variates. Permutation test was performed 1000 times for each trait in each population to determine the *P*-value when false discovery rate (FDR) equals to 0.05, as shown in the Manhattan plots and Table S7C. For some traits, permutation test obtained a -log_10_ *P*-value threshold that is too high and led to the loss of critical known QTLs (for example, GWAS for heading date trait with top three PCs as co-variates). To alleviate this type of false negative, we set the following criteria for screening candidate QTLs from EMMAX results: (1) SNPs with -log_10_ *P*-value passing the threshold of FDR < 0.05 both with and without top 3 PCs matrix as covariates; (2) SNPs with -log_10_ *P*-value passing FDR < 0.05 in one method and -log10 P-value passing 5 in another method; (3) SNPs with -log_10_ *P*-value passing 5 in at least one of the two methods and overlapping known QTL of that trait. We also performed GWAS with a new method, IIIVmrMLM (49), with default parameters.

**References**

52. IRRI. Standard Evaluation System for Rice (SES). International Rice Research Institute, Manila.; 2002.

53. Nagata, K, Ando, T, Nonoue, Y, et al. Advanced backcross QTL analysis reveals complicated genetic control of rice grain shape in a japonica x indica cross. Breed Sci. 2015; 65(4): 308-18.

54. Kawahara, Y, de la Bastide, M, Hamilton, JP, et al. Improvement of the Oryza sativa Nipponbare reference genome using next generation sequence and optical map data. Rice. 2013; 6: 4.

55. Li, H, Durbin, R. Fast and accurate short read alignment with Burrows-Wheeler transform. Bioinformatics. 2009; 25(14): 1754-60.

56. Li, H, Handsaker, B, Wysoker, A, et al. The Sequence Alignment/Map format and SAMtools. Bioinformatics. 2009; 25(16): 2078-9.

57. DePristo, MA, Banks, E, Poplin, R, et al. A framework for variation discovery and genotyping using next-generation DNA sequencing data. Nat Genet. 2011; 43(5): 491-8.

58. Alexander, DH, Novembre, J, Lange, K. Fast model-based estimation of ancestry in unrelated individuals. Genome Res. 2009; 19(9): 1655-64.

59. Reich, D, Price, AL, Patterson, N. Principal component analysis of genetic data. Nat Genet. 2008; 40(5): 491-2.

60. Paradis, E, Schliep, K. ape 5.0: an environment for modern phylogenetics and evolutionary analyses in R. Bioinformatics. 2019; 35(3): 526-8.

61. Malinsky, M, Matschiner, M, Svardal, H. Dsuite - Fast D-statistics and related admixture evidence from VCF files. Mol Ecol Resour. 2021; 21(2): 584-95.

62. Danecek, P, Auton, A, Abecasis, G, et al. The variant call format and VCFtools. Bioinformatics. 2011; 27(15): 2156-8.

63. Li, X, Chen, Z, Zhang, G, et al. Analysis of genetic architecture and favorable allele usage of agronomic traits in a large collection of Chinese rice accessions. Sci China Life Sci. 2020; 63(11): 1688-702.

64. Wei, X, Qiu, J, Yong, K, et al. A quantitative genomics map of rice provides genetic insights and guides breeding. Nat Genet. 2021; 53(2): 243-53.

65. Qin, P, Lu, H, Du, H, et al. Pan-genome analysis of 33 genetically diverse rice accessions reveals hidden genomic variations. Cell. 2021; 184(13): 3542-58 e16.

66. Kang, HM, Sul, JH, Service, SK, et al. Variance component model to account for sample structure in genome-wide association studies. Nat Genet. 2010; 42(4): 348-54.
